# p18 encoded by FgGMTV1 is responsible for asymptomatic infection in *Fusarium graminearum*

**DOI:** 10.1128/mbio.03066-24

**Published:** 2024-11-25

**Authors:** Lihang Zhang, Pengfei Li, Yanfei Wang, Shuangchao Wang, Lihua Guo

**Affiliations:** 1 State Key Laboratory for Biology of Plant Diseases and Insect Pests, Institute of Plant Protection, Chinese Academy of Agricultural Sciences, Beijing, China; 2 Guangdong Province Key Laboratory of Microbial Signals and Disease Control, College of Plant Protection, South China Agricultural University, Guangzhou, China; National Institutes of Health, Bethesda, Maryland, USA

**Keywords:** mycovirus, FgGMTV1, p18, *Fusarium graminearum*, hypovirulence, asymptomatic infection

## Abstract

The intricate interplay between mycoviruses and their fungal hosts frequently culminates in asymptomatic infections, but the virus-derived factors underlying these infections remain poorly understood. Our study introduces p18, a novel protein encoded by the DNA-C segment of the genomovirus FgGMTV1, which facilitates the transition from virus-induced hypovirulence to asymptomatic infection within *Fusarium graminearum* upon its expression. We have confirmed the expression of p18 during FgGMTV1 infection and observed its presence in both the nucleus and cytoplasm. Remarkably, strains with a p18 null mutation show a significant reduction in colony expansion, conidial production, and virulence, leading to a hypovirulent phenotype. Our results also indicate that p18 hinders the accumulation of FgGMTV1, thus determining asymptomatic infection and enabling vertical transmission through conidia. Furthermore, the p18 null mutant virus converts *F. graminearum* from virulent to hypovirulent strains on wheat leaves after horizontal transmission. This work not only expands our knowledge of the genomovirus proteome but also provides insights into the strategies of viral evolution and adaptation. Moreover, we propose an innovative approach for creating hypovirulent strains utilizing engineered mycoviruses for the biocontrol of plant pathogenic fungi.

**IMPORTANCE:**

Mycovirus-fungus interplay often leads to asymptomatic infections. Our study identifies p18, a novel protein from the genomovirus FgGMTV1, as a key determinant of asymptomatic infection in *Fusarium graminearum*. A p18-null mutant exhibits a pronounced hypovirulent phenotype. By modulating viral accumulation, p18 promotes asymptomatic infection and facilitates vertical transmission via conidia. This insight deepens our understanding of mycovirus-fungus interactions and introduces a novel strategy for biocontrol using engineered mycoviruses.

## INTRODUCTION

The interactions between viruses and their hosts can result in an array of diverse outcomes, encompassing potential benefits to the host, asymptomatic infections, varying degrees of disease severity, and even death (1). Notably, although numerous viruses undergo acute infections in host organisms, a vast array of viruses exist in an asymptomatic state, enabling them to replicate and spread systematically in the host without significantly affecting the host’s overall health (2, 3). In both humans and plants, asymptomatic viruses occasionally convert into acute infections, posing substantial challenges to public health or serious threats to cultivated crops (4–6). Hence, the underlying mechanisms of asymptomatic infections have garnered extensive and rigorous investigation across both human and plant virology. Mycoviruses, or fungal viruses, are ubiquitous across all major fungal taxonomic groups, frequently associated with asymptomatic infections (7). The hypovirulence (attenuation of fungal virulence) caused by a few mycoviruses, such as reduced pathogenicity, growth irregularity, abnormal pigmentation, and sexual development defects, have the potential to be used as biocontrol agents (BCAs) against plant pathogenic fungi (8). A classical example of BCA is the mycovirus Cryphonectria hypovirus 1 (CHV1), which effectively mitigates chestnut blight caused by *Cryphonectria parasitica* in Europe (9, 10). Recently, Sclerotinia sclerotiorum hypovirulence-associated DNA virus 1 (SsHADV-1) was successfully developed into a BCA, transforming the fungal host lifestyle from pathogenic to endophytic (11–13). Despite the discovery of hypovirulence-inducing mycoviruses in various plant pathogenic fungi, including *Fusarium spp*., *S. sclerotiorum*, and *Botrytis cinerea*, among others (14–17), the number of documented cases of mycovirus-induced hypovirulence remains a mere fraction compared with the hundreds of asymptomatic infection viruses that exhibit no discernible impact on their hosts (18). Therefore, it is important to investigate the key factors that contribute to asymptomatic infection and subsequently apply this knowledge to devise mycovirus-based BCAs.

Circular replication-associated protein-encoding single-stranded (CRESS) DNA viruses have been extensively documented in various environmental, fungal, plant, and animal samples (19). Presently, these viruses are systematically classified into 13 distinct families (20, 21). Notably, members of the *Geminiviridae* and *Nanoviridae* families are known to infect plants, acting as significant phytopathogens (22, 23). The functional roles of the proteins encoded by these viral members have been experimentally validated in numerous studies (24). Specifically, the tomato yellow leaf curl virus (TYLCV) genome harbors nine recognized open reading frames (ORFs), with the viral strand encoding V1 (coat protein, CP) and V2 (RNA silencing suppressor), whereas the complementary strand encodes C1 (replication-related Protein, Rep), C2 (transcription activator protein, TrAP), C3 (replication-enhancing protein, REn), and C4 (symptom determinant) (25). Moreover, TYLCV has been reported to encode additional small proteins (≤10 kDa), with three ORFs (V3, C5, and C7) being identified and functionally characterized (26–28). Fungal CRESS DNA viruses have only one family: *Genomoviridae*, which includes viruses with a small circular single-stranded (ss) DNA genome (ranging from ~1.8 to 2.4 kb), encoding a Rep and CP (29). The pioneering member of this family is SsHADV-1, followed by reports of the tripartite *Fusarium graminearum* gemytripvirus 1 (FgGMTV1), the tetra-segmented *Botrytis cinerea* ssDNA virus 1 (BcssDV1), *Botrytis cinerea gemydayirivirus* 1 (BcGDV1), and *Diaporthe sojae circular* DNA virus 1 (DsCDV1) (11, 30–33). Notably, SsHADV-1, BcGDV1, and DsCDV1 are monopartite viruses, all of which induce hypovirulence in their respective hosts. Conversely, the tri-segmented DNA virus FgGMTV1 exhibits the capacity to elicit asymptomatic infection or hypovirulence, dependent on its individual components (30). These recent discoveries of fungal CRESS DNA viruses have greatly promoted our understanding of mycovirus diversity and evolution. However, up until now, the functions of the proteins encoded by fungal CRESS DNA viruses have yet to be experimentally verified. Furthermore, our understanding of the mechanisms through which they modify fungal virulence remains quite limited.

The tripartite CRESS DNA mycovirus, FgGMTV1, was identified in *F. graminearum*, the causative agent of Fusarium head blight (FHB) in wheat (30). FgGMTV1 belongs to a new species, *Gemytripvirus fugra1*, within the family *Genomoviridae* (29). This virus comprises three genomic segments: DNA-A, encoding a Rep protein; DNA-B, encoding a CP protein; and DNA-C, encoding a protein p26 with an undefined function (30). The replication of DNA-A and DNA-B is mutually interdependent, leading to reduced growth and hypovirulence in their fungal hosts. Co-infection with all three segments, DNA-A, DNA-B, and DNA-C, results in viral asymptomatic infection, suggesting that the DNA-C component plays a pivotal role in regulating asymptomatic infection or hypovirulence. Moreover, DNA-C is indispensable for viral transmission through conidia (30). Nevertheless, the precise origins, coding sequences, and functionalities of the proteome encoded by DNA-C remain obscure.

Herein, our in-depth examination of the DNA-C segment of FgGMTV1 uncovers p18, a pivotal mediator of asymptomatic infection in *F. graminearum*. Notably, the absence of p18 results in induction of hypovirulence, underscoring its pivotal role. p18 not only facilitates the establishment of asymptomatic infection but also ensures viral persistence via vertical transmission through conidia. These findings deepen our understanding of viral-fungal interactions and opens avenues for creating hypovirulent strains to combat plant pathogens using engineered mycoviruses.

## RESULTS

### The FgGMTV1 genome encoded a novel p18 protein

**Fig 1:**
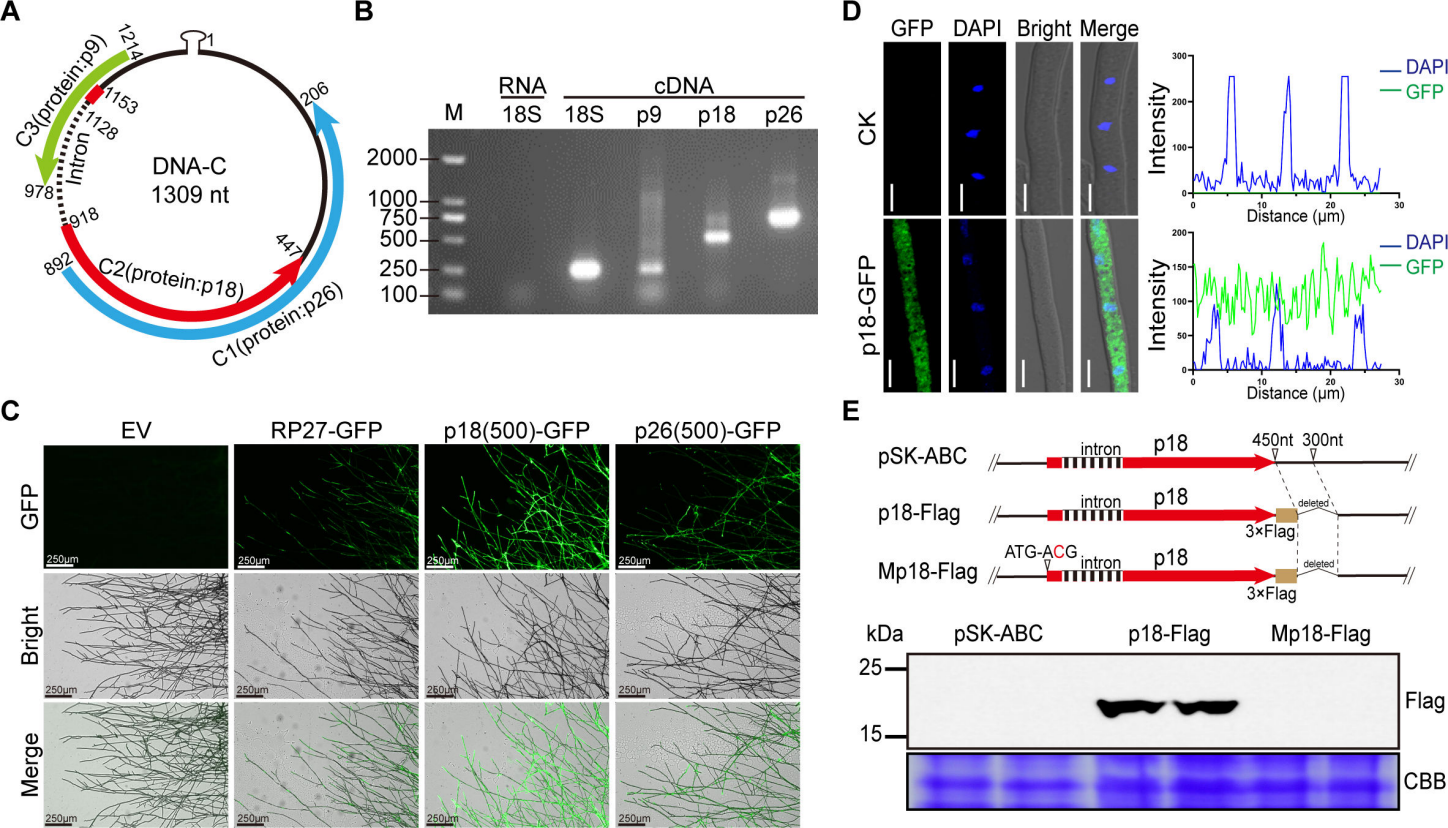
Novel open reading frames in the FgGMTV1 DNA-C genome and transcript detection of p18. (**A**) Genome organization of DNA-C of FgGMTV1; arrows indicate ORFs. The green arrow represents p9 protein, the red arrow represents p18 protein, and the blue arrow represents p26 protein. (**B**) RT-PCR analysis of p9, p18, and p26 transcripts from pSK-ABC-infected strains. M: DNA ladder marker. Negative control: PCR of total RNA extracted from pSK-ABC-infected strains using *Fg18S*-specific primers; Positive control: reverse transcription of total RNA extracted from pSK-ABC-infected strains using *Fg18S*-specific primers; p9, p18, and p26: reverse transcription of total RNA extracted from FgGMTV1-infected strains with p9, p18, or p26-specific primers, respectively. This experiment was repeated three times with similar results; representative images are shown. (**C**) The activity of p18 and p26 promoters (with the RP27 promoter as a positive control) in promoter-GFP fusion expression in *F. graminearum* PH-1 strains. Scale bar: 250 µm. EV: empty vector. This experiment was repeated three times with similar results; representative images are shown. (**D**) Subcellular localization of the OE-p18-GFP fusion protein determined by confocal microscopy (left panel). EV: empty vector. Bars: 5 µm. Nuclear localization was confirmed by simultaneous nuclear staining with 4′-6-diamidino-2-phenylinodle (DAPI). The colocalization of proteins was further evaluated by line-scan graph analysis (right panel). (**E**) Western blotting analysis of total protein from the p18-Flag and Mp18-Flag-infected strains using anti-Flag antibodies. Diagrams of the mutants p18-Flag and Mp18-Flag are shown in the upper panel. The mutant p18-Flag could express the p18 protein fused with a 3×Flag tag. Mp18-Flag was a site-directed mutagenesis of p18-Flag carrying a T1152C substitution in the p18 ATG (changing p18: Met to Thr and p9: Asn to Asn), impairing the production of the p18 protein and has no effect on the p9 protein. Total protein from pSK-ABC-infected strains was used as a negative control. Coomassie brilliant blue (CBB) staining shows equal loading. This experiment was repeated three times with similar results.

In a previous study, we demonstrated that DNA-A and DNA-B are related to hypovirulence of the fungal host, and DNA-C could counteract it (30). To explore the asymptomatic factors encoded by DNA-C genome, we utilized OFRfinder from NCBI (https://www.ncbi.nlm.nih.gov/orffinder/), Softberry (http://www.softberry.com/berry.phtml), and Augustus (http://bioinf.uni-greifswald.de/augustus/) to predict novel ORFs in DNA-C genomes. The identification revealed three ORFs (specifically, ORF C1, C2, and C3) on the complementary-sense (C) strand, each encoding proteins with an estimated molecular weight of approximately 10 kilodaltons (kDa) or larger (Fig. 1A). For further analyses, the positions of these three ORFs in the DNA-C genome is illustrated in Fig. 1A. Specifically, ORF C1 is situated at nucleotide positions 892–206, potentially encoding protein p26 of 228 amino acids with a molecular mass of 25.8 kDa (30). In contrast, ORF C2, which spans nucleotide positions 1153–447, incorporates an intron sequence located at nucleotide positions 1128–918. This unique ORF possesses the coding capacity for a protein designated as p18, comprising 165 amino acids with a molecular mass of 18.9 kDa. Finally, ORF C3, located at nucleotide positions 1214–978, has the potential to encode a shorter protein, named p9, composed of 78 amino acids with a molecular mass of 8.6 kDa.

A crucial prerequisite for p9, p18, and p26 to exhibit biological functionality is their expression within the context of viral infection. To ascertain the transcription of these proteins during viral infection, total RNA was extracted from the FgGMTV1-infected strain, which was derived through transfection of *F. graminearum* PH-1 with pSK-ABC, a previously established viral infectious clone that integrates the three components of FgGMTV1 into a non-integrated vector, pBluescript II SK (+)(pSK) (34). This RNA was then subjected to RT-PCR assays using p9-, p18-, or p26-specific primers (Table S1). As depicted in Fig. 1B, the existence of RNAs mapping to the specific ORFs of p9, p18, and p26 was confirmed in the pSK-ABC-infected strain. To further validate the expression of p9, p18, and p26, we cloned the −500 bp DNA-C genomic sequences upstream of the ATG start codons for both p18 and p26 and fused these sequences with an enhanced green fluorescent protein (eGFP) reporter gene, resulting in the construction of two recombinant vectors: p18(500 bp)-GFP and p26(500 bp)-GFP. Additionally, a positive control vector containing the ribosomal protein 27 promoter (RP27 promoter) fused with eGFP (RP27-GFP) was also prepared. These vectors were then transformed into the *F. graminearum* strain PH-1(WT) (Table S2). The promoter activity was quantitatively assessed through confocal microscopy by measuring the intensity of GFP fluorescence emitted. As illustrated in Fig. 1C, both −500 bp sequences could effectively activate the expression of GFP, leading to observable GFP fluorescence. Interestingly, compared with the RP27 promoter, the p18 promoter sequence displayed strong activity in driving the expression of GFP (Fig. 1C). These findings indicate that the −500 bp region upstream of both p18 and p26 possesses promoter activity, thereby demonstrating that p18, and p26 are expressed within their genomic context in *F. graminearum*. Furthermore, given the significant overlap between the promoter sequences of p9 and p18, it is plausible to assume that p9 is also expressed and functions accordingly.

In previous studies, we constructed p26 deletion viral mutants, p26-D1 to D3, in which 150 nucleotides (nt 4–153 for p26-D1, 154–303 for p26-D2, and 304–453 for p26-D3) were sequentially deleted with the p26 coding orientation. Due to infection with these viral deletion mutants, severe colony morphological abnormalities were observed, accompanied by a marked elevation in viral accumulation, in contrast to pSK-ABC-transfected strains (34). Notably, the deleted nucleotides in mutants p26-D1 to D3 align with p18 nucleic acid sequences. Conversely, mutant p26-D4-infected strains exhibited stable viral persistence and asymptomatic colony morphology (34), suggesting a potential influence of p18 on viral infection. Given that protein function is intricately tied to its subcellular localization, we utilized the PEG-mediated protoplast transformation method to express the p18-GFP fusion protein in the PH-1(WT) strain under the control of the RP27 promoter, thereby generating the OE-p18-GFP strain (Table S2). As depicted in Fig. 1D, p18 was found to be localized in both the nucleus and cytoplasm. To corroborate the expression of p18 in FgGMTV1-infected strains, we employed mass spectrometry to analyze pSK-ABC-transfected strains after 4 days of culturing. Our findings revealed the presence of four unique p18 polypeptides in FgGMTV1-infected strains, as detailed in Table S3. Furthermore, to gain further insights, we constructed viral mutants p18-Flag and Mp18-Flag, wherein the C-terminus of p18 was fused with a 3 × Flag tag, enabling the detection of p18 expression via western blotting using antibodies specific to Flag tags during viral infection (Fig. 1E). Notably, the mutant Mp18-Flag harbors a T1152C substitution in the p18 ATG start codon (p18: Met →Thr, p9: Asn→Asn), which disrupts the production of the p18 protein while sparing the p9 protein during viral infection (Fig. 1E). Utilizing PEG-mediated protoplast transfection, we generated p18-Flag and Mp18-Flag infected strains (Fig. S1; Table S2). Subsequently, total protein was extracted from these strains, with pSK-ABC-infected strains serving as a negative control. Western blotting analysis revealed distinct bands at 18 kDa in p18-Flag-infected strains, whereas no such bands were detected in pSK-ABC and Mp18-Flag-infected strains (Fig. 1E), thereby confirming the successful expression and detection of p18.

### p18 determines asymptomatic virus infection in *F. graminearum*

To evaluate the biological significance of the p9, p18, and p26 proteins in FgGMTV1 infection, we conducted site-directed mutagenesis on the viral infectious clone pSK-ABC to modify the initiation codon (ATG) of these proteins (Fig. S2). Specifically, Mp9 harbors a G1212C substitution within the p9 ATG (p9: Met→Ile), hindering the production of the p9 protein. Mp18 bears a T1152C substitution in the p18 ATG (p18: Met→Thr), disrupting the synthesis of the p18 protein without affecting p9. Similarly, Mp26 incorporates a T891C substitution in the p26 ATG (p26: Met→Thr), impairing p26 production while leaving p18 unaffected. Additionally, we generated Mp9:p18, Mp9:p26, Mp18:p26, and Mp9:p18:p26 mutants by altering the start codons (ATG) of both p9 and p18, p9 and p26, p18 and p26, and all three proteins, respectively, following a similar strategy (Fig. S2). These mutated constructs, Mp9, Mp18, Mp26, Mp9:p18, Mp9:p26, Mp18:p26, and Mp9:p18:p26, were then introduced into the *F. graminearum* strain PH-1(WT) via PEG-mediated protoplast transfection. For comparison, pSK-ABC- and pSK-AB-infected strains served as positive controls, whereas the virus-free strain PH-1 (WT) acted as the negative control. pSK-AB was generated by inserting 1.3-mer tandem repeats of DNA-A and DNA-B from FgGMTV1 into the pSK vector, employing a construction strategy similar to pSK-ABC, but excluding the incorporation of a 1.5-mer tandem repeat of DNA-C (Table S2).

**Fig 2:**
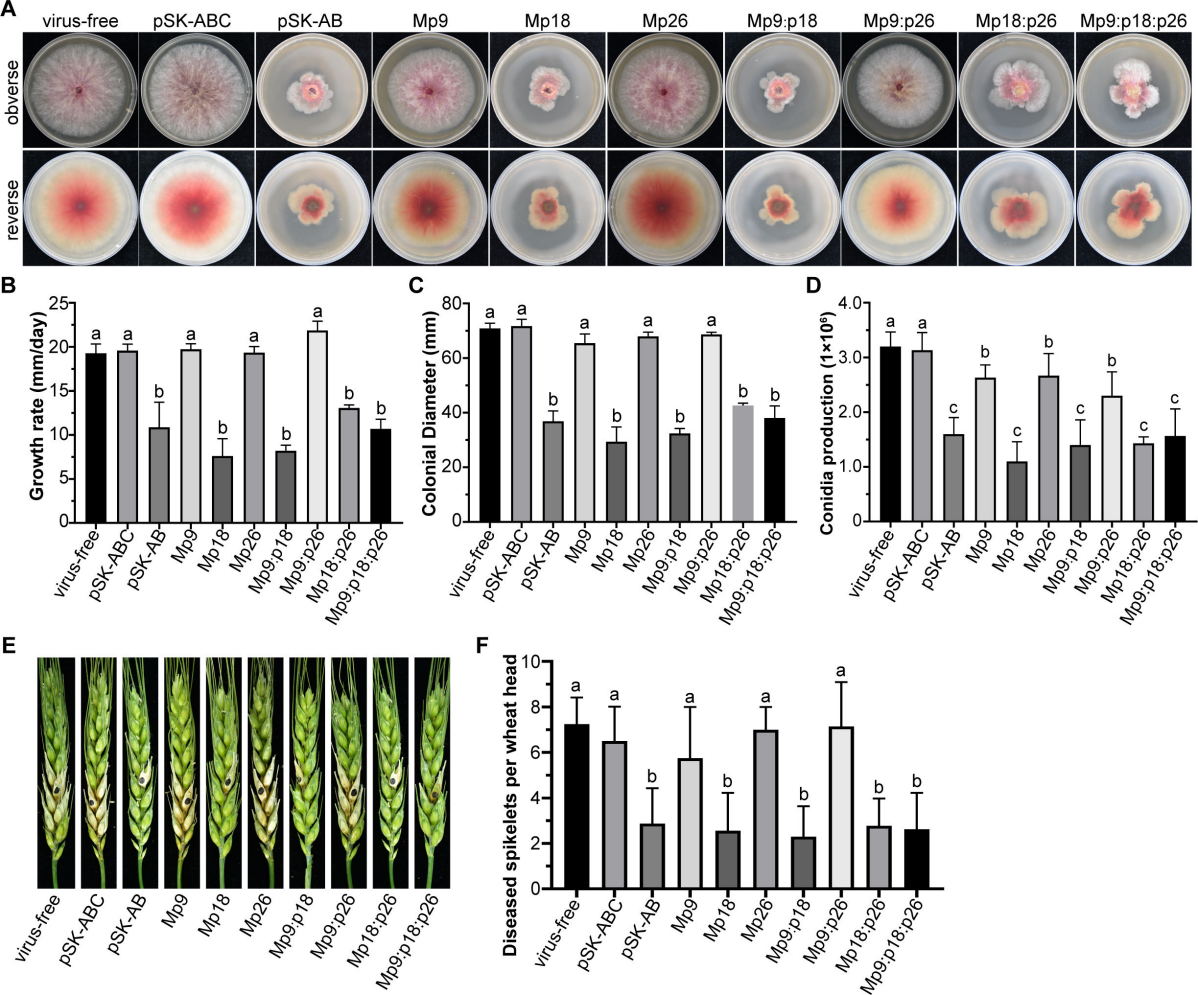
Comparison of colony morphology, colonial diameter, growth rate, conidial production, and virulence among PH-1(VF), pSK-AB, and pSK-ABC-infected strains, and seven different transfectants. (**A**) Colony morphology of PH-1 (VF), pSK-ABC, pSK-AB-infected strains, and the seven mutants after 4 days of culture on PDA in the dark (*n* = 3). (**B**) Growth rate of fungal strains on PDA medium (*n* = 3). (**C**) Comparison of colonial diameter among the samples from (**B**) (*n* = 3). (**D**) Conidia production after 5 days in CMC liquid medium (*n* = 3). (**E**) FHB symptoms were caused by PH-1(VF), pSK-ABC, pSK-AB-infected strains, and the seven mutants. (**F**) Number of diseased spikelets per invaded wheat head in sample (**A**). The number of diseased spikelets per invaded wheat head was counted at 12 dpi (*n* = 10). Error bars represent standard deviation. Different letters (a, b, and c) denote significant differences (*P* < 0.05 determined by Tukey’s *post hoc* test).

The study examines the impact of Mp9, Mp18, and Mp26, along with their combinations (Mp9:p18, Mp9:p26, Mp18:p26, Mp9:p18:p26) on the colony morphology, diameter, growth rate, and conidial production in *F. graminearum*. Following 4 days of cultivation, it was observed that Mp9, Mp26, and Mp9:p26-infected strains exhibited characteristics akin to those of pSK-ABC-infected strains and the virus-free PH-1(WT) strain (Fig. 2A). Conversely, Mp18, Mp9:p18, Mp18:p26, and Mp9:p18:p26-infected strains manifested similar abnormal colony morphologies to those infected with pSK-AB (Fig. 2A). Specifically, the growth rates of Mp18, Mp9:p18, Mp18:p26, and Mp9:p18:p26-infected strains on potato dextrose agar (PDA) were 7.6 mm/d, 8.2 mm/d, 13.1 mm/d, and 10.7 mm/d, respectively, markedly slower than those of PH-1 (19.3 mm/d) and pSK-ABC-infected strains (19.5 mm/d), and comparable with pSK-AB-infected strains (10.8 mm/d) (Fig. 2B). Furthermore, these strains demonstrated reductions in colony diameter of 59.1%, 54.8%, 41.6%, and 47.0%, respectively, compared with pSK-ABC-infected strains (Fig. 2C). Concurrently, a significant decline in conidial production was noted among Mp9, Mp18, Mp26, Mp9:p18, Mp9:p26, Mp18:p26, and Mp9:p18:p26-infected strains, with reductions of 16.1%, 64.9%, 14.9%, 55.3%, 26.6%, 54.3%, and 50%, respectively, compared with pSK-ABC-infected strains (Fig. 2D). Notably, the p18 protein emerged as the most crucial factor influencing conidial production, albeit with indications that p9 and p26 may also modulate this process. Collectively, these findings suggest that p18 attenuates the virulence of FgGMTV1 and plays a pivotal role in its asymptomatic infection.

Hypovirulent fungal strains hold promise as biological control agents against plant fungal diseases. To assess the impact of Mp18 on its fungal host, we conducted pathogenicity tests on flowering wheat heads. In this assay, small equalized mycelial plugs were inoculated into the glume of individual spikelets and subsequently cultivated in the field for 12 days. The Mp9, Mp26, and Mp9:p26-infected strains, as well as the virus-free strain PH-1(WT) and the pSK-ABC-infected strain, exhibited pronounced disease progression, spreading from the inoculated spikelets to others within the head, resulting in severe scab symptoms (Fig. 2E). In contrast, the Mp18, Mp9:p18, Mp18:p26, and Mp9:p18:p26-infected strains displayed a slower spread, with symptoms largely confined to the inoculated or immediately adjacent spikelets (Fig. 2E). Notably, their pathogenicity was significantly reduced by 60.7%, 64.6%, 57.3%, and 60.1%, respectively (Fig. 2F). Furthermore, the colony morphology of the p18-Flag and Mp18-Flag infected strains resembled that of the pSK-ABC and Mp18-infected strains, respectively (Fig. S1A). These findings suggest that the FgGMTV1 virus, when deprived of its p18 function, results in hypovirulence to pathogenic fungi, thereby highlighting its potential as a biological control agent.

We further developed p18-overexpressing strains (OE-p18-Flag) in the PH-1(WT) background and conducted complementation assays. Notably, the expression of the p18 protein was confirmed via western blotting analysis (Fig. S3). Importantly, the OE-p18-Flag strains did not exhibit any apparent developmental abnormalities (Fig. S4). As depicted in Fig. S4, overexpression of p18-Flag partially complemented the function of p18 when viral mutants Mp18 and pSK-AB infected the host. The colony morphologies indicated that strains PH-1(WT)::pSK-AB and PH-1(WT)::Mp18 produced either absent or sparse aerial mycelium, accompanied by a relatively slow growth rate. In contrast, strains OE-p18-Flag::pSK-AB and OE-p18-Flag::Mp18 displayed well-developed aerial mycelium and normal colony morphologies (Fig. S4A). Additionally, the colony diameters of strains OE-p18-Flag::pSK-AB and OE-p18-Flag::Mp18 were significantly larger than those of strains PH-1(WT)::pSK-AB and PH-1(WT)::Mp18 (Fig. S4B and C), demonstrating effective complementation.

### p18 plays a critical role in the vertical transmission of FgGMTV1

**Fig 3:**
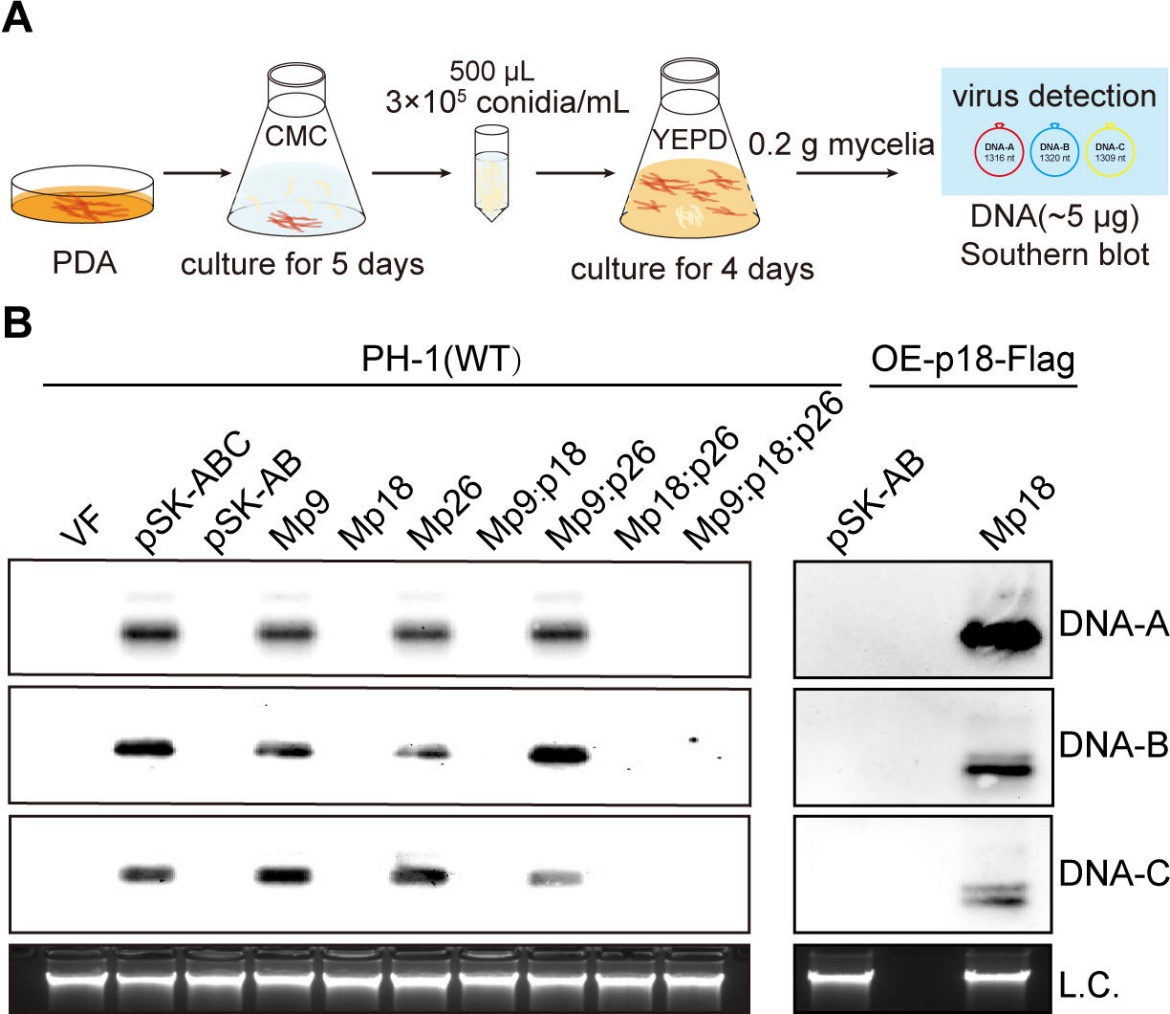
The capacity of the mutant viral vertical transmission through conidia. (**A**) Detection model for vertical transmission of viruses via conidia. Fresh conidia were cultured in arboxymethyl cellulose (CMC) liquid medium for 5 days. Five aliquots (500 µL) of the conidial suspensions, containing approximately 3 × 10^5^ conidia/mL, were inoculated into 50 mL of YEPD broth and cultured for 4 days at 25°C on a rotary shaker. Germinated mycelia (~0.2 g) were harvested, and total DNA (~5 µg) was extracted for Southern blotting analysis. (**B**) The blots were probed with probe A, probe B, and probe C, respectively. Fungal genomic DNA serves as the loading control (L.C.). This experiment was repeated three times with similar results; representative images are shown.

In our previous study, we established that the transmission of FgGMTV1 via conidia necessitates a DNA-C segment (30). Consequently, we examined the vertical transmission competency of DNA-C mutants. Specifically, five aliquots (500 µL) of conidial suspensions, each containing approximately 3 × 10^5^ conidia/mL, were inoculated into 50 mL of YEPD broth and cultured for 4 days at 25°C on a rotary shaker. The germinated mycelia were then harvested, and approximately 5 µg of total DNA was extracted for Southern blotting analysis (Fig. 3A). The findings revealed that viruses originating from pSK-ABC, Mp9, Mp26, and Mp9:p26-infected strains could be efficiently transmitted through conidia. Conversely, no viruses were detected in conidia produced by strains infected with pSK-AB, Mp18, Mp9:p18, Mp18:p26, or Mp9:p18:p26 (Fig. 3B). In addition, virus detection was absent in all single spore isolates derived from Mp18-infected strains, yielding zero infections among the 112 tested isolates. Conversely, the virus harbored in strains infected with pSK-ABC undergoes vertical transmission through conidia at a rate of 13.6%, which aligns with previously reported rates (30) (Fig. S5A). Furthermore, Southern blotting analyses confirmed efficient transmission of viruses via conidia from the OE-p18-Flag::Mp18 strain, in which the p18-transgenic strain OE-p18-Flag was infected by the viral mutant Mp18. Conversely, no viruses were detected in conidia derived from the OE-p18-Flag::pSK-AB strain, representing the p18-transgenic strain OE-p18-Flag infected by the viral mutant pSK-AB (Fig. 3B; Fig. S5B). This demonstrates that overexpressing p18 can compensate for the lack of viral transmission via conidia, suggesting that p18 is indispensable for the vertical transmission of FgGMTV1.

### p18 inhibits the accumulation of FgGMTV1

**Fig 4:**
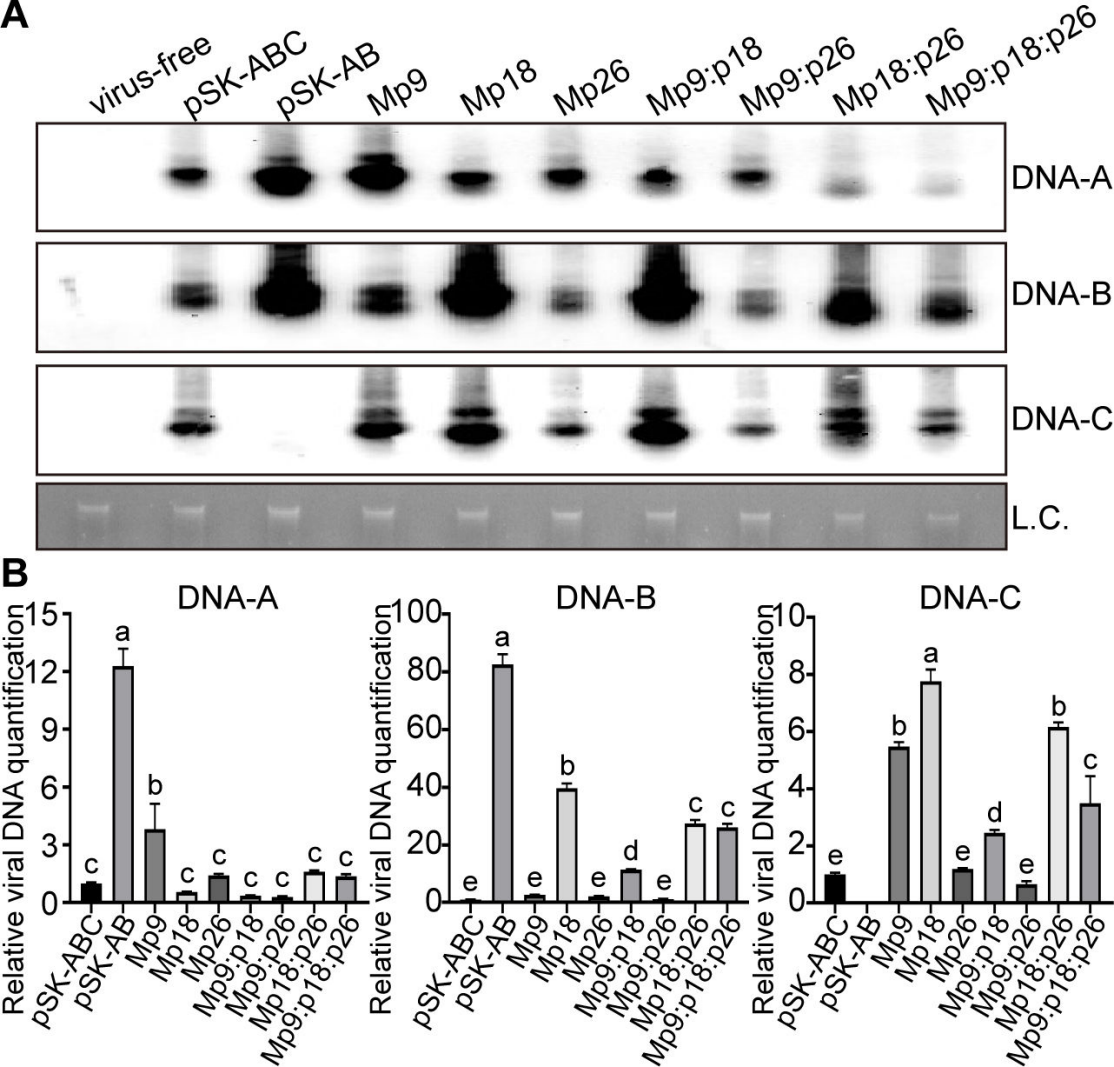
The viral genome accumulation levels were analyzed using Southern blotting and qPCR. (**A**) Southern blotting analysis was performed on DNA extracted from mycelia of PH-1 (virus-free, serving as a negative control), pSK-ABC, pSK-AB-infected strain (serving as a positive control), and the seven mutants. The DNA-A, DNA-B, and DNA-C components were detected using probe A, probe B, and probe C, respectively. Fungal genomic DNA was used as the loading control (L.C.). This experiment was repeated three times with similar results; representative images are shown. (**B**) The viral genome accumulation of DNA-A, DNA-B, and DNA-C components was determined by a quantitative PCR (qPCR) assay. Genomic DNA was extracted from strains cultured for 4 days. qPCR was performed using *EF-1α* as a reference. This experiment was repeated three times with similar results. Representative images are shown. Error bars represent standard deviation. Different letters denote significant differences (*P* < 0.05 determined by Tukey’s *post hoc* test).

To elucidate whether p18’s role in the asymptomatic infection and vertical transmission of FgGMTV1 is linked to viral accumulation, we conducted a Southern blotting analysis to quantify viral DNA accumulation across the seven mutant strains. Our findings revealed that strains infected with Mp18, Mp9:p18, and Mp18:p26 exhibited a pronounced enrichment of DNA-B and DNA-C components, in stark contrast to those infected with pSK-ABC (Fig. 4A). Intriguingly, Mp9-infected strains exhibited a marked enrichment of DNA-A and DNA-C components, whereas Mp26 and Mp9:p26-infected strains displayed no significant quantitative changes in virus accumulation compared with pSK-ABC-infected strains (Fig. 4A). These observations were corroborated by qPCR analysis, which mirrored the trends observed in the Southern blotting results (Fig. 4B). Furthermore, Southern blotting analysis indicated a decrease in the enrichment of DNA-A and DNA-B components in strains OE-p18-Flag::pSK-AB relative to PH-1(WT)::pSK-AB, and a significant reduction in the accumulation of DNA-B component specifically in the OE-p18-Flag::Mp18 strain compared with PH-1(WT)::Mp18 (Fig. S6). Collectively, these results underscore the pivotal role of p18 in inhibiting FgGMTV1 accumulation, thereby facilitating its asymptomatic infection and vertical transmission.

### Biocontrol potential of Mp18-induced hypovirulent strains against virulent *F. graminearum* strains

**Fig 5:**
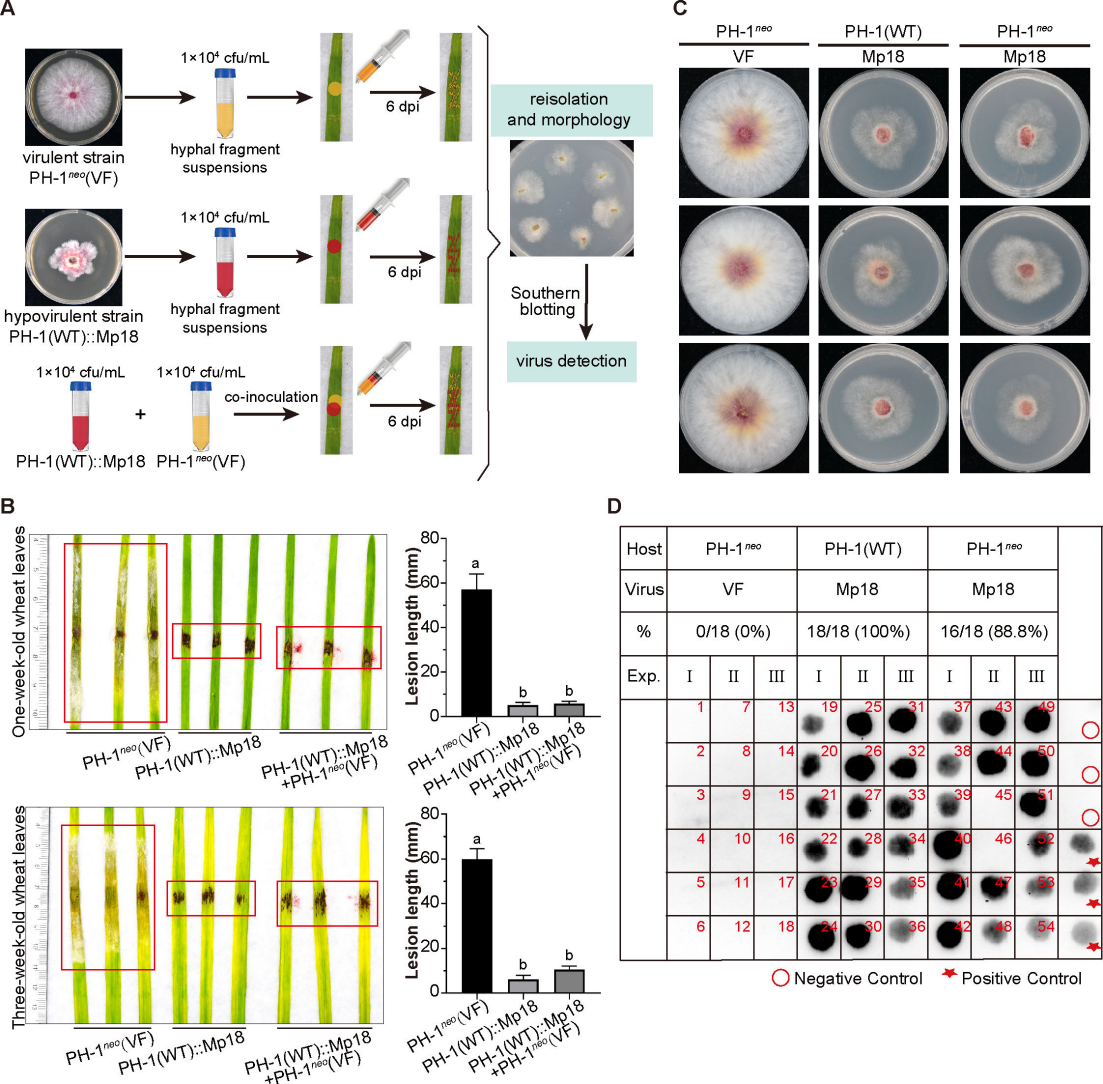
Biocontrol potential of Mp18-induced hypovirulent strains against virulent *F. graminearum* strains. (**A**) The diagram outlines the experimental procedure for assessing the biocontrol potential exhibited by strains infected with Mp18. Specifically, hyphal suspensions of approximately 1 × 10^4^ cfu/mL were inoculated onto the detached leaves from both 1- and 3-week-old wheat plants, with the virulent strain PH-1*^neo^*(VF) and with hypovirulent strains induced by Mp18, respectively. Additionally, Mp18-infected hyphal suspensions (≈1 × 10^4^ cfu/mL) were inoculated together with hyphal suspensions (≈1 × 10^4^ cfu/mL) of virulent strain PH-1*^neo^*(VF) (PH-1 incorporating a neomycin-resistance gene [*neo*]) on both 1- and 3-week-old wheat leaves. (**B**) Representative *F. graminearum* lesions and bar graph of lesion size on the leaves resulting from the aforementioned experimental procedure (*n* = 6). Fungal disease lesions were photographed 6 days after inoculation. Error bars represent standard deviation. Different letters denote significant differences (*P* < 0.05 determined by Tukey’s *post hoc* test). (**C**) Re-isolation of fungi from the disease lesions on leaves. Colony morphologies of representative reisolated *F. graminearum* strains with Mp18 infection or without Mp18 infection. The fungi were photographed at 3 days after culturing on PDA. (**D**) Dot Southern blotting detection of viral mutants Mp18 in the reisolated strains described in (**C**). This detection was conducted on reisolated strains after undergoing at least two subcultures. Dot Southern blotting analysis was performed with probe C. Red circles and stars indicate negative (PH-1, VF) and positive controls (pSK-ABC-infected strain), respectively.

Previous studies have highlighted the promising potential of mycovirus-mediated hypovirulence in managing fungal diseases (8). To further evaluate the efficacy of Mp18-infected strains in controlling the virulent strain PH-1, we performed an experiment where hyphal fragment suspensions of both Mp18-infected strain and virulent strain PH-1*^neo^*(VF), a derivative of PH-1 incorporating a neomycin-resistance gene (neo), were co-inoculated onto detached leaves from 1- and 3-week-old wheat plants. As control groups, we included inoculations with the virulent strain PH-1*^neo^*(VF) and with the hypovirulent strain harboring Mp18, respectively (Fig. 5A). After 6 days, the lesions that developed on wheat leaves following co-inoculation with both hypovirulent and virulent strains were significantly smaller (82.2% and 89.5% reduction on 1- and 3-week-old wheat leaves, respectively, *P* < 0.05) compared with those observed when only the virulent strain PH-1*^neo^* (VF) was inoculated. The lesion sizes in the co-inoculation group were comparable with those observed when only the hypovirulent strain was inoculated. Conversely, inoculation with the virulent strain PH-1*^neo^* (VF) alone resulted in the manifestation of extensive scab symptoms on wheat leaves (Fig. 5B). These results indicate that the hypovirulent strain induced by Mp18 holds promise as a biocontrol agent.

We also re-isolated the fungi from the infected leaves, evaluated their neomycin resistance, and confirmed the presence of Mp18 using dot Southern blotting (Fig. 5A). Accordingly, 16 of 18 re-isolated fungal strains of PH-1*^neo^* were found to be infected with Mp18, which showed slower growth on PDA medium compared with PH-1*^neo^*(VF) fungal strains, comparable with Mp18-infected PH-1(WT) strains (Fig. 5C). These observations indicate that re-isolated PH-1*^neo^* fungal strains acquire the viral mutants Mp18 from hypovirulent strains, and cross-infection with Mp18 suppresses the growth and pathogenicity of the virulent strain PH-1*^neo^* (Fig. 5C and D). Notably, all re-isolated PH-1(WT) strains infected by Mp18 retained the virus Mp18 with a 0% loss rate (Fig. 5C and D). These results suggest that the viral mutant Mp18 can be horizontally transmitted from hypovirulent to pathogenic strains under laboratory conditions. In conclusion, hypovirulent strains harboring the p18 null mutant effectively alleviate the virulence exhibited by the virulent strain of *F. graminearum* on wheat leaves through viral horizontal transmission.

## DISCUSSION

The investigation into viral gene products that promote asymptomatic infection has garnered substantial attention spanning various viral realms, including those infecting humans, animals, and plants. Nevertheless, the intricate panorama of asymptomatic factors in mycoviruses remains largely unexplored and enigmatic. In this study, we have pinpointed a novel protein, p18, residing within the DNA-C segment of the genomovirus FgGMTV1. Notably, p18 exhibits minimal homology to any known sequences in current databases, thereby significantly expanding the proteomic repertoire of genomoviruses. Our findings further establish p18 as a crucial factor, transitioning fungal hosts from hypovirulent to virulent strains through modulation of FgGMTV1 accumulation. This study not only sheds light on the intricate interplay between mycoviruses and their fungal hosts but also underscores the significance of p18 as a novel determinant of viral asymptomatic infection. This work represents a significant step forward in advancing our understanding of genomovirus biology and has the potential to inform future strategies for managing fungal diseases.

The reports have unveiled that geminiviral genomes harbor additional conserved ORFs, exhibiting specific subcellular localization patterns and diverse virulence functions, thereby expanding the repertoire of geminiviral proteins (23, 26). Recently, mycoviruses with multipartite DNA genomes have garnered attention, with FgGMTV1 comprising three circular ssDNA segments (DNA-A, DNA-B, and DNA-C) and BcssDV1 consisting of four circular ssDNA segments (DNA-A, DNA-B, DNA-C, and DNA-D) (30, 31, 35). Notably, the *F. graminearum*/FgGMTV1 system serves as an invaluable model for investigating viral asymptomatic infections and virus-host interactions (30). In this study, we have demonstrated that the FgGMTV1 DNA-C segment harbors additional ORFs, devoid of any discernible sequence homology with known entries in public databases, apart from previously characterized ORFs (Fig. 1A). We have singled out three larger ORFs (ORF C1, C2, and C3) on the complementary-sense strand, with their respective protein products designated as p26 (as described in reference 30), p18, and p9, for in-depth scrutiny. Our findings confirm that p9, p18, and p26 are expressed during FgGMTV1 infection, with the existence of the p18 protein being further verified through western blotting (Fig. 1B, C, and E). Intriguingly, the p18 protein primarily localizes to the nucleus and cytoplasm of the host cell (Fig. 1D). Remarkably, the p18 null mutant resulted in hypovirulence to the pathogenic fungus *F. graminearum* (Fig. 2A through F). Thus, elucidating the functions of the proteins encoded by the FgGMTV1 DNA-C component enriches the proteome of genomoviruses.

Mycoviruses that induce hypovirulence (attenuation of fungal virulence) have garnered significant attention as promising agents for the biological control of fungal diseases (36). Nevertheless, the majority of viruses infecting economically significant plant pathogenic fungi remain ineffective in influencing host growth, dissemination, or pathogenicity. To address this, virus-induced gene silencing (VIGS) vectors based on FgGMTV1 have been developed to generate hypovirulent strains aimed at controlling fusarium head blight (FHB) (34). In our current study, we present a p18 null mutant, Mp18, which uniquely harbors a T1152C substitution within the p18 ATG start codon, transforming the asymptomatic state of the pathogenic fungus *F. graminearum* into a hypovirulent phenotype. This Mp18-infected strain holds promise as a BCA against wheat scab. Consequently, we propose an innovative approach leveraging modified mycoviruses to create hypovirulent strains, offering a novel biocontrol strategy against plant pathogenic fungi.

The virus infection process is exceedingly intricate, encompassing diverse mechanisms to sustain infection. In the context of fungi-mycovirus interactions, RNA silencing or RNA interference (RNAi) serves primarily as an antiviral defense mechanism (37). Conversely, viral suppressors of RNA silencing (VSR) utilize a myriad of strategies to inhibit these RNA silencing pathways (38). Furthermore, research has illuminated that the fungal host’s RNAi machinery undergoes downregulation in the presence of myco-VSR, thereby facilitating viral infection (39–42). For instance, the papain-like protease p29, encoded by hypovirus CHV1-EP713, functions as a myco-VSR to enhance the accumulation of genomic RNA (39). In our study, the DNA-B components displayed notably increased accumulation in both pSK-AB-infected strains and p18 null mutant virus-infected strains (Fig. 4A). Concurrently, pSK-AB-infected strains and Mp18-infected strains exhibited diminished mycelial growth and severely aberrant colony morphology on PDA culture media (Fig. 2A). Notably, overexpression of the p18 protein results in downregulation of DNA-B accumulation and partially restores the abnormal fungal phenotypes (Fig. S6). Therefore, it is hypothesized that DNA-B may encode virulence factors, whereas p18 acts as a pivotal factor regulating viral accumulation and infection.

Furthermore, we delved into the role of p18 in the vertical transmission of FgGMTV1. Prior research revealed that FgGMTV1 fails to propagate through conidia in the absence of DNA-C (30). Our current findings echo this notion, demonstrating that the p18 null mutant Mp18 is similarly incapable of transmission via conidia (Fig. 3). Notably, although overexpression of the p18 protein restores the viral transmission capacity of Mp18, it does not facilitate the dissemination of the virus pSK-AB through asexual spores (Fig. 3B). This underscores the necessity of DNA-C-encoded p18 for FgGMTV1’s transmission via conidia, albeit DNA-C likely encodes additional factors that bolster viral transmission. In the field of mycoviruses, their primary modes of transmission encompass intracellular dissemination through hyphal anastomosis (horizontal transmission) or sporulation (sexual or asexual, vertical transmission) (36). Over millennia, viruses and their hosts have engaged in an evolutionary arms race, necessitating adaptations that ensure host survival while facilitating viral spread. For instance, in the CHV1-*Cryphonectria parasitica* system, reduced viral genomic RNA accumulation leads to decreased transmission through conidia. Notably, CHV1-encoded p29 acts as an enhancer for both viral RNA accumulation and vertical transmission via asexual spores (43). However, excessive viral accumulation often elicits robust antiviral immune responses, threatening host health. In response, plant viruses have devised multifaceted strategies to manipulate host defenses, thereby enhancing insect vector proliferation and disease dissemination (23, 44). For example, TYLCV modulates the Janus kinase/signal transducer and activator of transcription (JAK/STAT) signaling pathway to restrict viral accumulation while maintaining transmission in whiteflies (45). Consequently, the evolution of DNA and RNA viruses exhibits intricate mechanisms that balance viral propagation with host homeostasis.

Moreover, the viral infection process is highly intricate, encompassing alterations in host gene expression patterns, reprogramming of signaling controls, disruption of central cellular metabolic pathways, impairment of the host’s defense mechanisms, and the adept evasion of RNA silencing responses, thereby enhancing host susceptibility (23). Our findings suggest that p18 harbors an N-glycosylation site, a protein kinase C phosphorylation site, a casein kinase II phosphorylation site, an N-myristoylation site, and a microbodies C-terminal targeting signal (Fig. S7). Recent studies have underscored the pivotal role of these protein modifications or pathways in influencing diverse stages of viral infection or antiviral immunity (46–49). Furthermore, proteome and transcriptome analyses have affirmed that proteins and genes modulated by mycovirus infection are implicated in the growth, development, and stress responses of fungal hosts (50, 51). In *F. graminearum*, several signal transduction pathways critical for developmental and metabolic processes have been investigated, including the cyclic adenosine monophosphate (cAMP)-protein kinase A (PKA) pathway, mitogen-activated protein kinase (MAPK) cascades, and the target of rapamycin (TOR) pathway (52). Herein, we observed that the cAMP-PKA and MAPK signaling pathways, epigenetic regulation, sporogenesis, calcium signaling, and other virulence and stress response-related genes were differentially regulated in Mp18-infected strains compared with pSK-ABC-infected strains and virus-free strains (Fig. S8). These results imply that p18 may function as a pivotal factor, interacting with essential host genes, thereby exerting influence over the growth, development, sexual and asexual sporulation, and as well as pathogenicity of *F. graminearum*. A more in-depth investigation into the p18 will foster a deeper comprehension of the evolutionary and ecological interplay between viruses and their hosts, subsequently enabling us to harness the full potential of mycoviruses more effectively.

## MATERIALS AND METHODS

### Fungal strains, plant materials, and culture conditions

All fungal strains used in this study are listed in Table S2 and were cultured on PDA medium for 3–5 days at 25°C for morphological observation or on cellophane-covered PDA medium for DNA and RNA extractions. Carboxymethyl cellulose (CMC) liquid medium was used for conidiation assays. Yeast extract peptone dextrose (YEPD) liquid medium was used to culture fungal strains for protein extractions. PH-1 served as the wild-type strain of *F. graminearum* (53).

The susceptible wheat cultivar, Yangmai158 was grown in the greenhouse under a 16 h light and 8 h dark cycle at 25°C for pathogenicity assays conducted on flowering wheat heads.

### Plasmid construction

To generate the constructs for the analysis of promoter activity, the 500-nt sequence upstream of the p18 and p26 ATG was PCR-amplified and cloned into the pGTN-GFP vector, which had been digested with *Kpn* I and *Not* I. The resulting constructs were named p18(500)-GFP and p26(500)-GFP, respectively. To generate the construct for expressing OE-p18-GFP, the full-length p18 ORF (with introns removed) was obtained by RT-PCR and subsequently recombined into the pGTN-GFP vector, which had been digested with *Hind* III and *Bam*H I. Furthermore, the full-length p18 gene sequence (with introns removed) was recombined into the pGTN-3×Flag vector, which was used to express the 3×Flag p18 protein.

Mutations were introduced into the p9, p18, and p26 genes of DNA-C of FgGMTV1 to investigate the function of these ORF. The mutation strategy is detailed in Fig. S2. Due to the presence of a 1.5-mer tandem repeat of DNA-C in the infectious clone pSK-ABC, the initiation codons (ATGs) of p9 and p18 were duplicated (Fig. S9). The mutations of p9 and p18 were constructed in a series of steps (Fig. S9). Initially, the plasmid pSK-ABC was digested with *Nco* I, and the obtained 1.5-mer tandem repeat of the DNA-C fragment, approximately 1.9 kb, was inserted into the *Nco* I site of the vector pBluescript II SK(+) (pSK) (Stratagene) to generate the clone pSK-C. Subsequently, the remaining ~5.5 kb vector fragment was self-ligated and named pSK-AB. The clone pSK-1C was then digested with *Hind* III, *Xba* I, and *Not* I, and the obtained fragments of approximately 1.28 kb and 0.65 kb were inserted into the vector pSK digested with *Hind* III/*Xba* I and *Xba* I/*Not* I, respectively, to produce the clone pSK-0.6C and pSK-0.4C. Site-directed mutagenesis of 0.6C-Mp9 and 0.4C-Mp9 was performed using complementary primer pairs with a KOD-Plus-Mutagenesis Kit (Toyobo, Osaka, Japan) based on the plasmids pSK-0.6C and pSK-0.4C. Next, the plasmid 0.4C-Mp9 was digested with *Hind* III and *Xba* I, and the obtained ~0.65 kb fragment was inserted into the *Hind* III and *Xba* I sites of the plasmid 0.6C-Mp9 to generate the clone C-Mp9. C-Mp9 was further linked back to the pSK-AB vector and named Mp9. Mp18 was constructed using a similar procedure as mentioned above. Mp26 was constructed following a similar strategy, but site-directed mutagenesis of C-Mp26 was performed based on pSK-C. Mp9:p18, Mp9:p26, Mp18:p26, and Mp9:p18:p26 were constructed using similar procedures as mentioned above.

To detect the expression of the p18 protein, we constructed the mutant p18-Flag, which expresses the p18 protein fused with a 3×Flag tag (Fig. 1E). This construct was derived from the previously described plasmid p26-D4. Additionally, Mp18-Flag was generated through site-directed mutagenesis of p18-Flag, which halted the expression of p18 using a similar procedure as mentioned above.

Primers used in this work are listed in Table S1, and all constructs were confirmed through sequencing.

### Sequence analysis and prediction of domains in protein sequences

The NCBI OFRfinder (https://www.ncbi.nlm.nih.gov/orffinder/), Softberry (http://www.softberry.com/berry.phtml), and Augustus (http://bioinf.uni-greifswald.de/augustus/) were used to identify ORFs within the DNA-C genomes.

### Protoplast preparation, transfection, and transformation

The preparation of protoplasts from *F. graminearum* strains was performed using a previously described method (30). The transfection of *F. graminearum* with viral infectious clones and viral mutants was accomplished utilizing the polyethylene glycol (PEG)-mediated protoplast transfection method, as previously detailed (30). For the transformation, 30 µg (~30 µL) of recombinant plasmids was mixed with 500 µL of protoplasts and incubated on ice for 30 min. Subsequently, 3.5 mL of PTC buffer (STC buffer containing 40% polyethylene glycol 4000) was added dropwise to the mixture of protoplasts and plasmids, followed by incubation on ice for another 30 min. After incubation, the protoplast suspension was transferred to 10 mL of TB3 liquid medium and incubated for 12 h at 25°C in a shaker at 120 rpm. The germlings were then transferred to 15 cm petri dishes, embedded in 20 mL of warm TB3 solid medium containing 200 mg/mL G418, and cultured for 12 h. Then, the upper culture medium (40 mL of warm TB3 solid medium containing 400 mg/mL G418) was added, and the cultures were incubated for 3–5 days at 25°C in the dark. Subsequently, transformants were selected on PDA medium supplemented with G418 and purified by single-spore isolation.

### DNA extraction, Southern blotting, and qPCR

Genomic DNA was extracted from fungi using the MolPure Plant DNA Kit (Yeasen) following the manufacturer’s instructions. Southern blotting was used to detect viral infections and viral accumulation. Probes A (359 bp fragments of DNA-A), B (335 bp fragments of DNA-B), and C (687 bp fragments of DNA-C), labeled with digoxigenin (DIG), were specifically used to detect the accumulation of DNA-A, DNA-B, and DNA-C components, respectively. Probe preparation, hybridization, and signal detection were performed using the DIG-High Prime DNA Labeling and Detection Starter Kit II (Roche, Basel, Switzerland). The primer sequences for generating the probes were described previously (30, 34). For the detection of viral DNA accumulation by qPCR, the fragments of DNA-A, DNA-B, and DNA-C were amplified. *FgEF-1α* was used as an internal reference for DNA normalization. The primer pairs used in this study are listed in Table S1.

### RNA extraction, RT-PCR, and RT-qPCR

Total RNA was extracted from the mycelium of fungi using the FastPure Universal Plant Total RNA Isolation Kit (Vazyme) according to the manufacturer’s instructions. The extracted RNA was dissolved in RNase-free water and stored at −80°C. Approximately 1 µg of total RNA was reverse-transcribed into cDNA using the Hifair III 1st Strand cDNA Synthesis Kit (Yeasen Biotechnology). PCR was performed using KOD-Plus-Neo (TOYOBO). Quantitative PCR (qPCR) was conducted on an ABI Prism 7500 PCR instrument using the SYBR Green Real-Time PCR Master Mix (Vazyme Biotech). Relative gene expression was calculated using the 2^-ΔCT^ method. *FgEF-1α* was used as an internal reference for RNA normalization. The primer pairs used in this study are listed in Table S1.

### Protein extraction and western blotting

Total protein was isolated from fresh mycelia (500 mg), which were finely ground and suspended in 1 mL of extraction buffer containing 10 µL of protease inhibitor cocktail (Sangon Co., Shanghai, China). Immunoblotting was performed using a mouse monoclonal anti-FLAG antibody (HT201-01, TransGen, 1:5000 dilution). Following inoculation with the secondary antibody, goat polyclonal anti-mouse IgG-HRP (HS201-01, TransGen, 1:5,000 dilution), chemiluminescence was detected.

### Observation of GFP fluorescence

For observation of GFP fluorescence intensity, sterile glass coverslips (22 × 22 mm) were placed on cellophane overlaying PDA, and a small mycelial plug was inserted at the edge of each coverslip. The hyphae of the transfected fungal colonies were allowed to grow onto the coverslips. Subsequently, the coverslips with attached hyphae were transferred to a glass slide, and GFP fluorescence was observed using a microscope (Carl Zeiss). For localization, vegetative hyphae of the strains were observed under a Zeiss LSM980 confocal microscope after incubation in YEPD medium at 25°C for 16 h. 4′,6-diamidino-2-phenylindole (DAPI) staining was used to visualize the nuclei.

### Mycelial growth, conidiation, and pathogenicity

Colony morphology and mycelial growth were assayed on PDA for 4 days at 25°C. The growth rate of fungi was calculated by measuring colonial diameters daily after culturing for 4 days. For conidiation assays, three agar plugs of each strain were inoculated in a 100 mL flask containing 50 mL of CMC liquid medium and cultured for 5 days. Subsequently, conidial production was determined on blood count plates. For the virulence assay, a small equalized mycelial plug was inoculated in the glume of a spikelet and cultured in the field for 12 days. At the end of the cultivation period, virulence was assessed by measuring the number of diseased spikelets per wheat head invaded. There were 10 replicates for each strain. The biocontrol assay is presented in Fig. 5A. Briefly, hyphal fragment suspensions containing approximately 1 × 10^4^ cfu/mL were inoculated onto the detached leaves from 1- to 3-week-old wheat plants. Six days post-inoculation, the disease lesions on each inoculated wheat leaf were documented and collected. There were six replicates for each strain.

### Viral vertical transmission through conidia

To assess the potential for vertical transmission of mutants via conidia, three aliquots (500 µL) of conidial suspensions, containing approximately 3 × 10^5^ conidia/mL, were inoculated into 50 mL of YEPD broth and cultured for 5 days at 25°C on a rotary shaker. Subsequently, germinated mycelia were harvested and analyzed for the presence of the mycovirus by performing total DNA purification and Southern blotting. Additionally, the potential for viral vertical transmission via conidia was investigated using germinated mycelia derived from conidial suspensions containing approximately 1 × 10^7^ conidia/mL, 1 × 10^5^ conidia/mL, and 1 × 10^3^ conidia/mL, respectively. To assess the frequency of viral vertical transmission, single conidia were isolated, and the resultant monoconidial cultures were analyzed for the presence of the mycovirus by performing total DNA purification and dot Southern blotting.

### Statistical analysis

Data are represented as the mean ± standard deviation (SD). Statistical analyses were performed using GraphPad Prism version 8.0. Significant differences between the control and treatment groups were analyzed using one-way ANOVA followed by the Dunnett test or two-way ANOVA by the Tukey test for multiple group comparisons. Values are shown as means ± standard deviation. Statistical significance was set at *P* < 0.05. The sample size (*n*) for each statistical analysis has been reported in the corresponding figure legends.

## SUPPLEMENTAL MATERIAL

Supplemental Material mbio.03066-24-s0001.pdf Figures S1 to S9; Tables S1 to S4.

ASM does not own the copyrights to Supplemental Material that may be linked to, or accessed through, an article. The authors have granted ASM a non-exclusive, world-wide license to publish the Supplemental Material files. Please contact the corresponding author directly for reuse.

## References

[1] Hillman BI, Annisa A, Suzuki N. 2018. Viruses of plant-interacting fungi. Adv Virus Res 100:99–116. doi:10.1016/bs.aivir.2017.10.003 29551145

[2] Takahashi H, Fukuhara T, Kitazawa H, Kormelink R. 2019. Virus latency and the impact on plants. Front Microbiol 10:2764. doi:10.3389/fmicb.2019.02764 31866963 PMC6908805

[3] Knipe DM, Raja P, Lee J. 2017. Viral gene products actively promote latent infection by epigenetic silencing mechanisms. Curr Opin Virol 23:68–74. doi:10.1016/j.coviro.2017.03.010 28415052 PMC5475406

[4] Ilyas R, Rohde MJ, Richert-Pöggeler KR, Ziebell H. 2022. To be seen or not to be seen: latent infection by tobamoviruses. Plants (Basel) 11:2166. doi:10.3390/plants11162166 36015469 PMC9415976

[5] Cary DC, Fujinaga K, Peterlin BM. 2016. Molecular mechanisms of HIV latency. J Clin Invest 126:448–454. doi:10.1172/JCI80565 26731470 PMC4731164

[6] Kanda T. 2018. EBV-encoded latent genes. Adv Exp Med Biol 1045:377–394. doi:10.1007/978-981-10-7230-7_17 29896676

[7] Ghabrial SA, Castón JR, Jiang D, Nibert ML, Suzuki N. 2015. 50-plus years of fungal viruses. Virol (Auckl) 479–480:356–368. doi:10.1016/j.virol.2015.02.034 25771805

[8] Wagemans J, Holtappels D, Vainio E, Rabiey M, Marzachì C, Herrero S, Ravanbakhsh M, Tebbe CC, Ogliastro M, Ayllón MA, Turina M. 2022. Going viral: virus-based biological control agents for plant protection. Annu Rev Phytopathol 60:21–42. doi:10.1146/annurev-phyto-021621-114208 35300520

[9] Nuss DL. 2005. Hypovirulence: mycoviruses at the fungal-plant interface. Nat Rev Microbiol 3:632–642. doi:10.1038/nrmicro1206 16064055

[10] Rigling D, Prospero S. 2018. Cryphonectria parasitica, the causal agent of chestnut blight: invasion history, population biology and disease control. Mol Plant Pathol 19:7–20. doi:10.1111/mpp.12542 28142223 PMC6638123

[11] Yu X, Li B, Fu YP, Jiang DH, Ghabrial SA, Li GQ, Peng YL, Xie JT, Cheng JS, Huang JB, Yi XH. 2010. A geminivirus-related DNA mycovirus that confers hypovirulence to a plant pathogenic fungus. Proc Natl Acad Sci U S A 107:8387–8392. doi:10.1073/pnas.0913535107 20404139 PMC2889581

[12] Zhang H, Xie J, Fu Y, Cheng J, Qu Z, Zhao Z, Cheng S, Chen T, Li B, Wang Q, Liu X, Tian B, Collinge DB, Jiang D. 2020. A 2-kb mycovirus converts a pathogenic fungus into a beneficial endophyte for brassica protection and yield enhancement. Mol Plant 13:1420–1433. doi:10.1016/j.molp.2020.08.016 32998002

[13] Tian B, Xie J, Fu Y, Cheng J, Li B, Chen T, Zhao Y, Gao Z, Yang P, Barbetti MJ, Tyler BM, Jiang D. 2020. A cosmopolitan fungal pathogen of dicots adopts an endophytic lifestyle on cereal crops and protects them from major fungal diseases. ISME J 14:3120–3135. doi:10.1038/s41396-020-00744-6 32814863 PMC7784893

[14] Li P, Bhattacharjee P, Wang S, Zhang L, Ahmed I, Guo L. 2019. Mycoviruses in Fusarium species: an update. Front Cell Infect Microbiol 9:257. doi:10.3389/fcimb.2019.00257 31380300 PMC6657619

[15] Sahin E, Akata I. 2018. Viruses infecting macrofungi. Virusdisease 29:1–18. doi:10.1007/s13337-018-0434-8 29607353 PMC5877858

[16] Kondo H, Kanematsu S, Suzuki N. 2013. Viruses of the white root rot fungus, Rosellinia necatrix. Adv Virus Res 86:177–214. doi:10.1016/B978-0-12-394315-6.00007-6 23498907

[17] Wang J, Quan R, He X, Fu Q, Tian S, Zhao L, Li S, Shi L, Li R, Chen B. 2023. Hypovirus infection induces proliferation and perturbs functions of mitochondria in the chestnut blight fungus. Front Microbiol 14:1206603. doi:10.3389/fmicb.2023.1206603 37448575 PMC10336323

[18] Bocos-Asenjo IT, Niño-Sánchez J, Ginésy M, Diez JJ. 2022. New insights on the integrated management of plant diseases by RNA strategies: mycoviruses and RNA interference. Int J Mol Sci 23:9236. doi:10.3390/ijms23169236 36012499 PMC9409477

[19] Zhao L, Rosario K, Breitbart M, Duffy S. 2019. Eukaryotic Circular Rep-Encoding Single-Stranded DNA (CRESS DNA) viruses: ubiquitous viruses with small genomes and a diverse host range. Adv Virus Res 103:71–133. doi:10.1016/bs.aivir.2018.10.001 30635078

[20] Desingu PA, Nagarajan K, Powell EA. 2022. Genetic diversity and characterization of Circular Replication (Rep)-Encoding Single-Stranded (CRESS) DNA viruses. Microbiol Spectr 10:e0105722. doi:10.1128/spectrum.01057-22 36346238 PMC9769708

[21] Krupovic M, Varsani A, Kazlauskas D, Breitbart M, Delwart E, Rosario K, Yutin N, Wolf YI, Harrach B, Zerbini FM, Dolja VV, Kuhn JH, Koonin EV. 2020. Cressdnaviricota: a virus phylum unifying seven families of Rep-encoding viruses with single-stranded, circular DNA genomes. J Virol 94:e00582-20. doi:10.1128/JVI.00582-20 32269128 PMC7307096

[22] Li F, Qiao R, Wang Z, Yang X, Zhou X. 2022. Occurrence and distribution of geminiviruses in China. Sci China Life Sci 65:1498–1503. doi:10.1007/s11427-022-2125-2 35661965

[23] Wu J, Zhang Y, Li F, Zhang X, Ye J, Wei T, Li Z, Tao X, Cui F, Wang X, Zhang L, Yan F, Li S, Liu Y, Li D, Zhou X, Li Y. 2024. Plant virology in the 21st century in China: recent advances and future directions. J Integr Plant Biol 66:579–622. doi:10.1111/jipb.13580 37924266

[24] Fiallo-Olivé E, Lett J-M, Martin DP, Roumagnac P, Varsani A, Zerbini FM, Navas-Castillo J. 2021. ICTV virus taxonomy profile: Geminiviridae 2021. J Gen Virol 102. doi:10.1099/jgv.0.001696 PMC8744271 34919512

[25] Li F, Qiao R, Yang X, Gong P, Zhou X. 2022. Occurrence, distribution, and management of tomato yellow leaf curl virus in China. Phytopathol Res 4. doi:10.1186/s42483-022-00133-1

[26] Gong P, Tan H, Zhao S, Li H, Liu H, Ma Y, Zhang X, Rong J, Fu X, Lozano-Durán R, Li F, Zhou X. 2021. Geminiviruses encode additional small proteins with specific subcellular localizations and virulence function. Nat Commun 12:4278. doi:10.1038/s41467-021-24617-4 34257307 PMC8277811

[27] Zhao S, Gong P, Ren Y, Liu H, Li H, Li F, Zhou X. 2022. The novel C5 protein from tomato yellow leaf curl virus is a virulence factor and suppressor of gene silencing. Stress Biol 2:19. doi:10.1007/s44154-022-00044-3 37676365 PMC10442036

[28] Liu H, Chang Z, Zhao S, Gong P, Zhang M, Lozano-Durán R, Yan H, Zhou X, Li F. 2023. Functional identification of a novel C7 protein of tomato yellow leaf curl virus. Virol (Auckl) 585:117–126. doi:10.1016/j.virol.2023.05.011 37331112

[29] Varsani A, Krupovic M. 2021. Family Genomoviridae: 2021 taxonomy update. Arch Virol 166:2911–2926. doi:10.1007/s00705-021-05183-y 34331585

[30] Li P, Wang S, Zhang L, Qiu D, Zhou X, Guo L. 2020. A tripartite ssDNA mycovirus from a plant pathogenic fungus is infectious as cloned DNA and purified virions. Sci Adv 6:eaay9634. doi:10.1126/sciadv.aay9634 32284975 PMC7138691

[31] Ruiz-Padilla A, Turina M, Ayllón MA. 2023. Molecular characterization of a tetra segmented ssDNA virus infecting Botrytis cinerea worldwide. Virol J 20:306. doi:10.1186/s12985-023-02256-z 38114992 PMC10731770

[32] Khalifa ME, MacDiarmid RM. 2021. A mechanically transmitted DNA mycovirus is targeted by the defence machinery of its host, Botrytis cinerea. Viruses 13:1315. doi:10.3390/v13071315 34372522 PMC8309985

[33] Wang X, Kotta-Loizou I, Coutts RHA, Deng H, Han Z, Hong N, Shafik K, Wang L, Guo Y, Yang M, Xu W, Wang G. 2024. A circular single-stranded DNA mycovirus infects plants and confers broad-spectrum fungal resistance. Mol Plant 17:955–971. doi:10.1016/j.molp.2024.05.003 38745413

[34] Zhang L, Wang S, Ruan S, Nzabanita C, Wang Y, Guo L. 2023. A mycovirus VIGS vector confers hypovirulence to a plant pathogenic fungus to control wheat FHB. Adv Sci (Weinh) 10:e2302606. doi:10.1002/advs.202302606 37587761 PMC10582431

[35] Ruiz-Padilla A, Rodríguez-Romero J, Gómez-Cid I, Pacifico D, Ayllón MA, Wickner RB. 2021. Novel mycoviruses discovered in the mycovirome of a necrotrophic fungus. mBio 12:e03705-20. doi:10.1128/mBio.03705-20 33975945 PMC8262958

[36] Myers JM, James TY. 2022. Mycoviruses. Curr Biol 32:R150–R155. doi:10.1016/j.cub.2022.01.049 35231405

[37] Zhao J-H, Guo H-S. 2022. RNA silencing: from discovery and elucidation to application and perspectives. J Integr Plant Biol 64:476–498. doi:10.1111/jipb.13213 34964265

[38] Hough B, Steenkamp E, Wingfield B, Read D. 2023. Fungal viruses unveiled: a comprehensive review of mycoviruses. Viruses 15:1202. doi:10.3390/v15051202 37243288 PMC10224137

[39] Segers GC, van Wezel R, Zhang X, Hong Y, Nuss DL. 2006. Hypovirus papain-like protease p29 suppresses RNA silencing in the natural fungal host and in a heterologous plant system. Eukaryot Cell 5:896–904. doi:10.1128/EC.00373-05 16757737 PMC1489278

[40] Yaegashi H, Yoshikawa N, Ito T, Kanematsu S. 2013. A mycoreovirus suppresses RNA silencing in the white root rot fungus, Rosellinia necatrix. Virol (Auckl) 444:409–416. doi:10.1016/j.virol.2013.07.010 23896640

[41] Aulia A, Hyodo K, Hisano S, Kondo H, Hillman BI, Suzuki N. 2021. Identification of an RNA silencing suppressor encoded by a symptomless fungal hypovirus, Cryphonectria hypovirus 4. Biol (Basel) 10:100. doi:10.3390/biology10020100 PMC7912522 33572564

[42] Yu J, Park JY, Heo JI, Kim KH. 2020. The ORF2 protein of Fusarium graminearum virus 1 suppresses the transcription of FgDICER2 and FgAGO1 to limit host antiviral defences. Mol Plant Pathol 21:230–243. doi:10.1111/mpp.12895 31815356 PMC6988435

[43] Suzuki N, Maruyama K, Moriyama M, Nuss DL. 2003. Hypovirus papain-like protease p29 functions in trans to enhance viral double-stranded RNA accumulation and vertical transmission. J Virol 77:11697–11707. doi:10.1128/jvi.77.21.11697-11707.2003 14557655 PMC229363

[44] Zhao S, Wu Y, Wu J. 2021. Arms race between rice and viruses: a review of viral and host factors. Curr Opin Virol 47:38–44. doi:10.1016/j.coviro.2021.01.002 33530035

[45] Wang Y-M, He Y-Z, Ye X-T, Guo T, Pan L-L, Liu S-S, Ng JCK, Wang X-W. 2022. A balance between vector survival and virus transmission is achieved through JAK/STAT signaling inhibition by a plant virus. Proc Natl Acad Sci U S A 119:e2122099119. doi:10.1073/pnas.2122099119 36191206 PMC9564230

[46] Ferreira AR, Marques M, Ramos B, Kagan JC, Ribeiro D. 2022. Emerging roles of peroxisomes in viral infections. Trends Cell Biol 32:124–139. doi:10.1016/j.tcb.2021.09.010 34696946

[47] Pandey VK, Sharma R, Prajapati GK, Mohanta TK, Mishra AK. 2022. N-glycosylation, a leading role in viral infection and immunity development. Mol Biol Rep 49:8109–8120. doi:10.1007/s11033-022-07359-4 35364718 PMC8974804

[48] Zhao X, Wang X, Dong K, Zhang Y, Hu Y, Zhang X, Chen Y, Wang X, Han C, Yu J, Li D. 2015. Phosphorylation of Beet black scorch virus coat protein by PKA is required for assembly and stability of virus particles. Sci Rep 5:11585. doi:10.1038/srep11585 26108567 PMC4479801

[49] Wang B, Dai T, Sun W, Wei Y, Ren J, Zhang L, Zhang M, Zhou F. 2021. Protein N-myristoylation: functions and mechanisms in control of innate immunity. Cell Mol Immunol 18:878–888. doi:10.1038/s41423-021-00663-2 33731917 PMC7966921

[50] Yu J, Kim KH. 2021. A phenome-wide association study of the effects of Fusarium graminearum transcription factors on Fusarium graminearum virus 1 infection. Front Microbiol 12:622261. doi:10.3389/fmicb.2021.622261 33643250 PMC7904688

[51] Gao Z, Wu J, Jiang D, Xie J, Cheng J, Lin Y. 2020. ORF Ι of mycovirus SsNSRV-1 is associated with debilitating symptoms of Sclerotinia sclerotiorum. Viruses 12:456. doi:10.3390/v12040456 32316519 PMC7232168

[52] Chen Y, Kistler HC, Ma Z. 2019. Fusarium graminearum trichothecene mycotoxins: biosynthesis, regulation, and management. Annu Rev Phytopathol 57:15–39. doi:10.1146/annurev-phyto-082718-100318 30893009

[53] Cuomo CA, Güldener U, Xu J-R, Trail F, Turgeon BG, Di Pietro A, Walton JD, Ma L-J, Baker SE, Rep M, et al.. 2007. The Fusarium graminearum genome reveals a link between localized polymorphism and pathogen specialization. Science 317:1400–1402. doi:10.1126/science.1143708 17823352

