# Supplementary material for: p18 encoded by FgGMTV1 is responsible for asymptomatic infection in Fusarium graminearum
Source: mBio. 2024 Nov 25;16(1):e03066-24. doi: 10.1128/mbio.03066-24 (PMC11708013; doi:10.1128/mbio.03066-24)
Supplement: Supplemental Material — Figures S1 to S9; Tables S1 to S4. [file mbio.03066-24-s0001.pdf]

Supporting Information for

p18 Encoded by FgGMTV1 is Responsible for Asymptomatic Infection in *Fusarium graminearum*

Lihang Zhang [a], Pengfei Li [b], Yanfei Wang [a], Shuangchao Wang [a], Lihua Guo [a, 1]

* Corresponding author: Lihua Guo[1]

Email: guolihua@caas.cn

This PDF file includes:

Figures S1 to S9
Tables S1 to S4
References 1-23

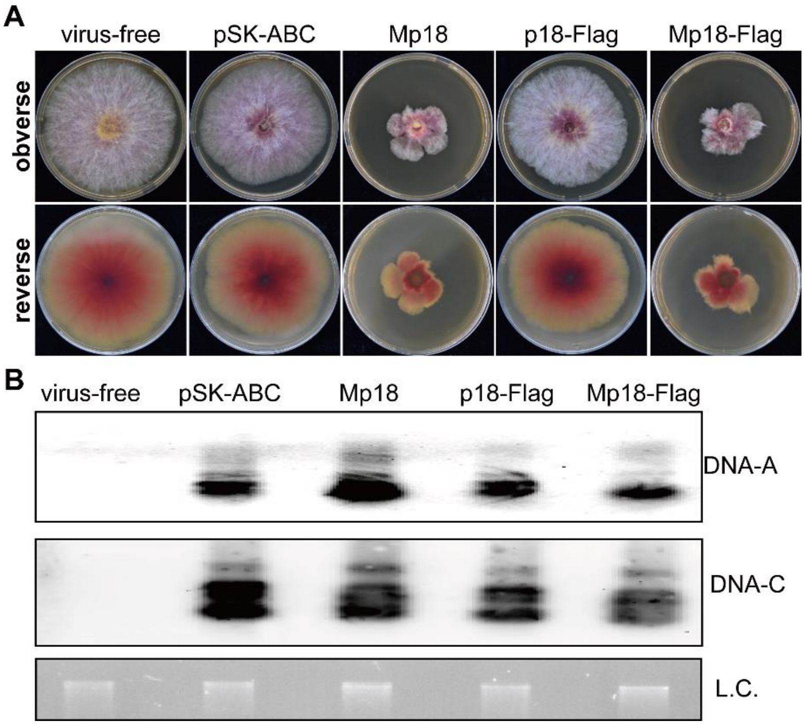

Fig. S1. Colony morphology and infection assessment of mutants p18-Flag and Mp18-Flag. (A) Colony morphology of the p18-Flag and Mp18-Flag infected strains. The fungal strains were cultured on PDA plates for 4 days (n = 3). (B) Southern blotting analysis of DNAs extracted from PH-1(WT), pSK-ABC, Mp18, p18-Flag, and Mp18-Flag infected strains. The DNA-A and DNA-C components were blotted with probe A and probe C, respectively. Fungal genomic DNA serves as the loading control (L.C.).

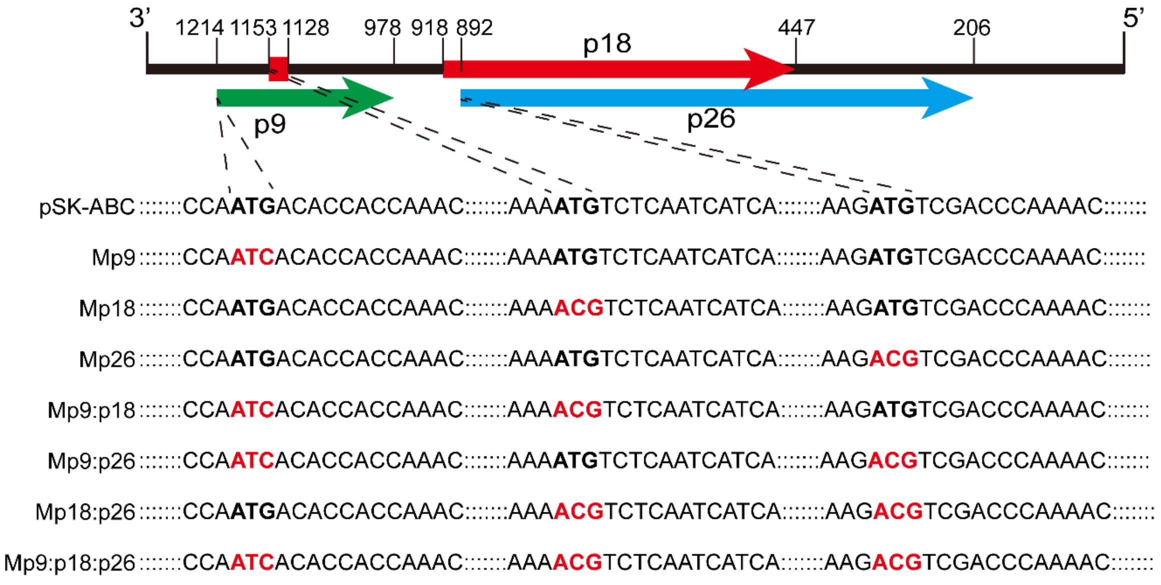

Fig. S2. Schematic representation of DNA-C and mutational strategies for the p9, p18, and p26 genes. The green arrow (1214-978) represents the protein p9, the red arrow (1153-1128, 918-447) represents protein p18, and the blue arrow(892-206) represents protein p26. Line 1 shows the wild-type sequences, with the start codons of p9, p18, and p26 genes highlighted in bold. The Mp9 mutant carries a G1212C substitution in the p9 ATG (p9: Met→Ile). The Mp18 mutant features a T1152C substitution in the p18 ATG (p18: Met→Thr, p9 unaffected: Asn→Asn). The Mp26 mutant incorporates a T891C substitution in the p26 ATG (p26: Met→Thr, p18 unaffected: Asp→Asp). Additionally, combinatorial mutations Mp9:p18, Mp9:p26, Mp18:p26, and Mp9:p18:p26 were generated by altering the start codon (ATG) of p9 and p18, p9 and p26, p18 and p26, or all three start codons (ATG) of p9, p18, and p26, respectively, using a similar approach.

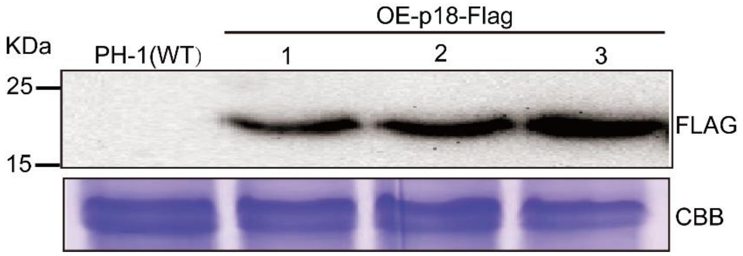

Fig. S3. Western blotting analysis of total protein extracted from p18-overexpression strains with anti-Flag antibodies. Total protein from the strain PH-1 serves as the negative control. Coomassie brilliant blue (CBB) staining confirms equal protein loading among the samples.

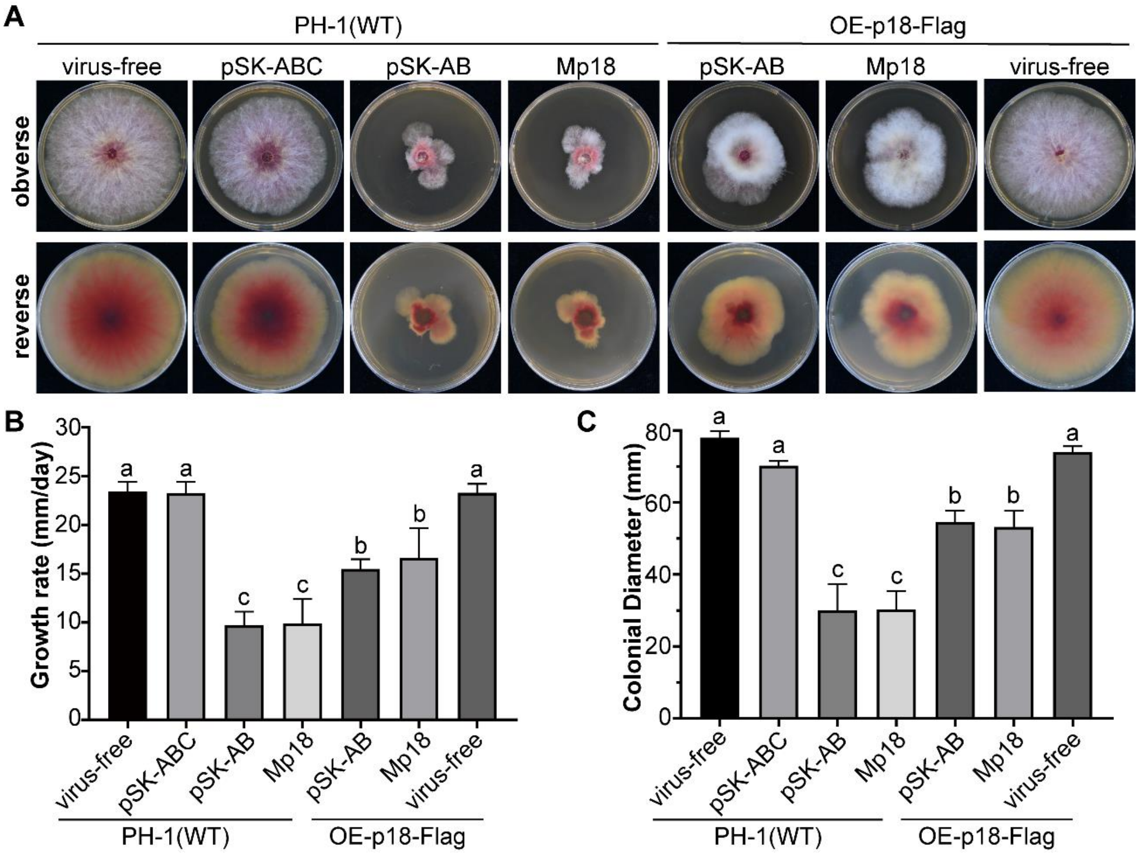

Fig. S4. Functional complementation of p18 overexpression in pSK-AB and Mp18 infected strains. (A) Colony morphology of strains PH-1(WT), PH-1(WT)::pSK-ABC, PH-1(WT)::pSK-AB, PH-1(WT)::Mp18, OE-p18-Flag, OE-p18-Flag::pSK-AB and OE-p18-Flag::Mp18. The fungal strains were cultured on PDA plates for 4 days (n = 3). (B) Growth rate of fungal strains on PDA medium (n = 3). (C) Comparison of colonial diameters among samples from (A) (n = 3). Error bars represent standard deviation. Different letters (a, b and c) denote significant differences ($p < 0.05$, determined by Tukey's post hoc test).

A

Efficiency of virus transmission through conidia

Virus strain	pSK-ABC	Mp18
Host strain	PH-1(WT)	
No. of infected /No. of tested	15/110	0/112
%	13.6	0

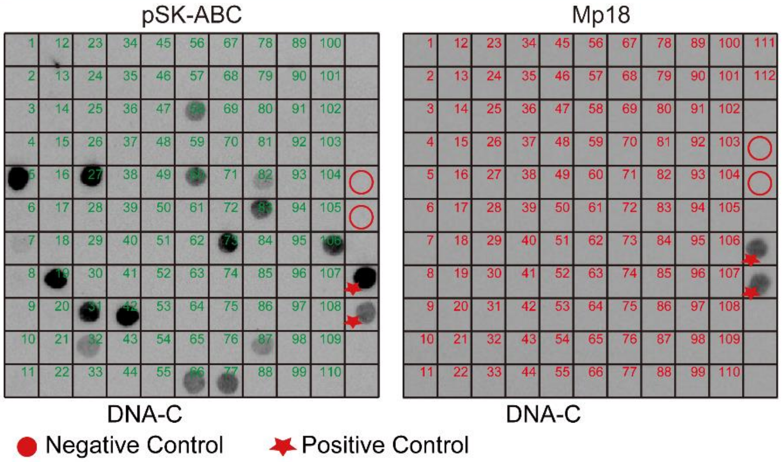

B

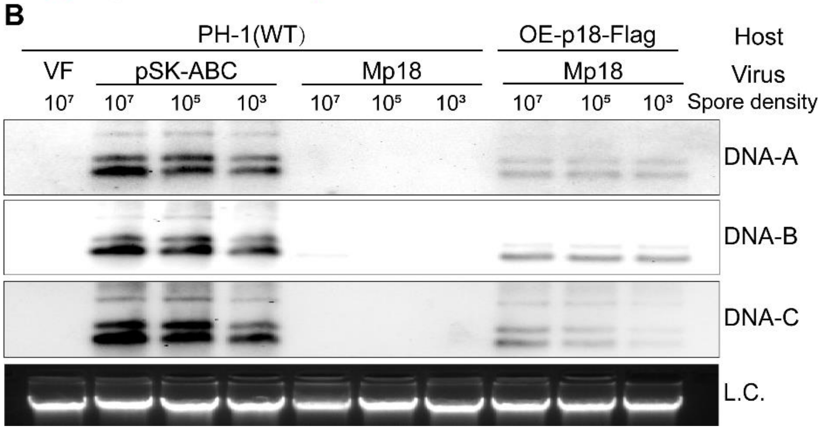

Fig. S5. Viral vertical transmission through conidia. (A) The efficiency of the viral mutant vertical transmission through conidia. Fresh conidia were cultured in CMC (carboxymethyl cellulose) liquid medium for 5 days. Single spore was picked and germinated for 4 days at 25 °C on PDA. All samples were derived from spores of pSK-ABC and Mp18-infected strains, and dot blotting analysis was performed with Probe C. Red circles and stars indicate negative (PH-1, VF) and positive controls (pSK-ABC-infected strain), respectively. (B) Vertical transmission analysis of viral mutants Mp18. Fresh conidia were cultured in CMC (carboxymethyl cellulose) liquid medium for 5 days. Five aliquots (500 µL) of the conidial suspensions, containing approximately 1×10^7 conidia/mL, 1×10^5 conidia/mL, and 1×10^3 conidia/mL, respectively, were inoculated into 50 mL of YEPD broth and cultured for 4 days at 25 °C on a rotary shaker. Germinated mycelia (~0.2 g) were harvested, and total DNA (~5 µg) was extracted for Southern blotting analysis. The blots were

probed with Probe A, Probe B, and Probe C, respectively. Fungal genomic DNA serves as the loading control (L.C.). This experiment was repeated three times with similar results. Representative images are shown.

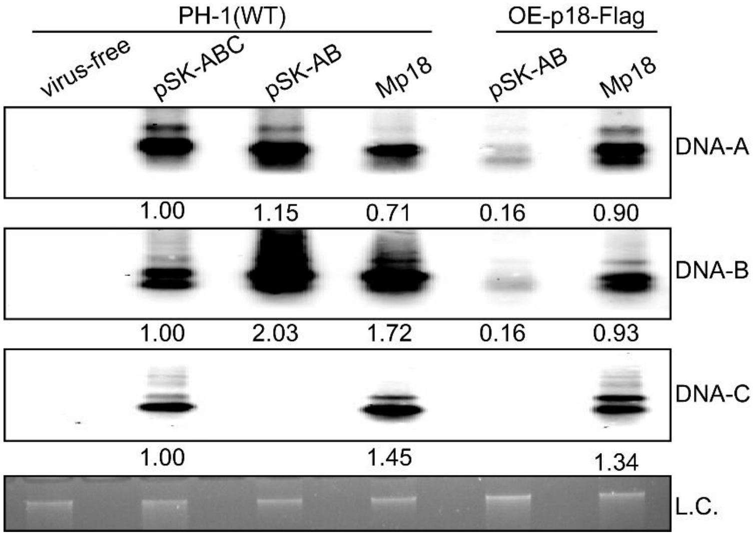

Fig. S6. Analysis of viral genome accumulation levels via Southern blotting. DNA extracted from various strains including OE-p18-Flag::pSK-AB, OE-p18-Flag::Mp18, PH-1(WT)::pSK-ABC, PH-1(WT)::pSK-AB and PH-1(WT), were subjected to Southern blotting analysis. The DNA-A, DNA-B, and DNA-C components were blotted with Probe A, Probe B, and Probe C, respectively. Fungal genomic DNA serves as the loading control (L.C.). The values of samples of PH-1(WT)::pSK-ABC were set as 1.00. This experiment was repeated three times with similar results. Representative images are shown.

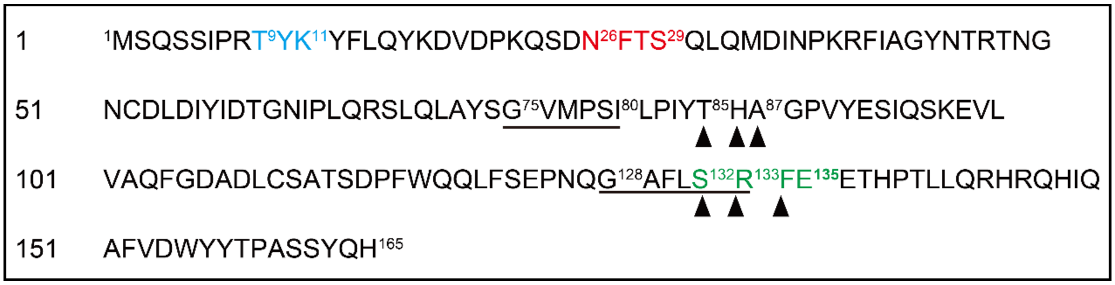

Fig. S7. Prediction of modification sites in the p18 protein. N-glycosylation site is presented in red. The Protein kinase C phosphorylation site is presented in blue. The Casein kinase II phosphorylation site is presented in green. The N-myristoylation site is presented with underline. The Microbodies C-terminal targeting signal is presented with triangle.

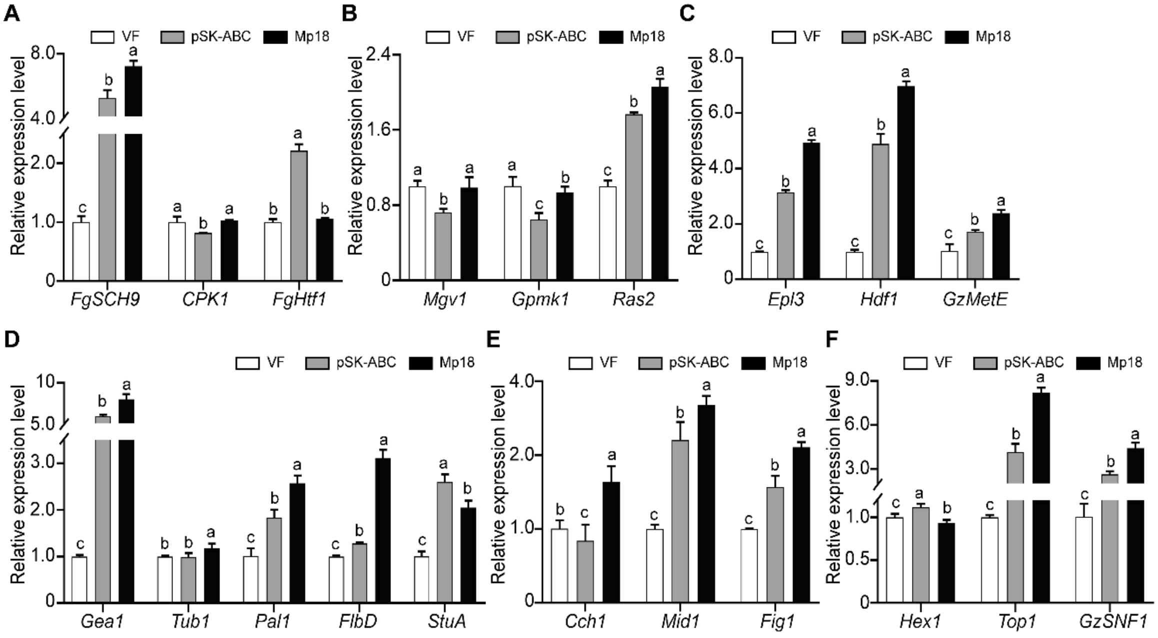

Fig. S8. p18 regulates the accumulation of transcripts from key host genes during viral asymptomatic infection. To gain deeper insights into the potential molecular mechanisms underlying virus-induced asymptomatic or hypovirulent infection in the host fungus, we conducted a comparative analysis of fungal gene expressions at the transcript level between pSK-ABC and Mp18-infected strains. These genes encompass diverse cellular processes, as detailed in Table S4(35-57). (A and B) In *F. graminearum*, several signal transduction pathways critical for developmental and metabolic processes have been investigated, including the cyclic adenosine monophosphate (cAMP)-protein kinase A (PKA) pathway, mitogen-activated protein kinase (MAPK) cascades, and the target of rapamycin (TOR) pathway. Subsequently, we zeroed in on three cAMP-PKA signaling pathway-associated genes (FgSCH9, FGSG_00472; CPK1, FGSG_07251; and FgHtf1, FGSG_07097) and three MAPK signaling pathway-related genes (Mgv1, FGSG 10313; Gpmk1, FGSG_06385; and Ras2, FGSG_10114) for further scrutiny. The RT-qPCR assay revealed a notable upregulation in the expression of five genes (FgSCH9, CPK1, Mgv1, Gpmk1, and Ras2) and a downregulation of FgHtf1 in Mp18-infected strains compared to pSK-ABC-infected strains. This finding underscores the modulatory role of p18 in transcription related to these signal transduction pathways. (C) To unravel the functional role of p18 in the epigenetic regulation of fungi, we examined the transcripts of three genes (Epl3, FGSG_02040; Hdf1, FGSG_01353; GzmetE, FGSG_05658) that are involved in covalent post-translational modifications (PTMs) of histones. These genes exhibited significant induction in Mp18-infected strains. These results collectively indicate that p18 might be intricately involved in epigenetic regulation, thereby facilitating asymptomatic infection. (D and E) Since p18 plays a pivotal role in vertical transmission mediated by conidia, we subsequently selected genes associated with spore development and discharge in fungi for validation. As depicted in Fig. S8, D and E, the introduction of Mp18 significantly upregulated the transcriptional activity of eight genes (Gea1, FGSG_08505; Tub1, FGSG 09530; FgPal1, FGSG_08726; FlbD, FGSG_01915; StuA, FGSG_10129; Cch1, FGSG_01364; FgMid1, FGSG_07418; and Fig1,

FGSG_06302), implying that the p18 protein modulates the processes of spore development and discharge. (F) We also identified three genes (Hex1, FGSG_08737; Top1, FGSG_06874; and GzSNF1, FGSG_09897) related to DNA repair and stress responses for analysis. Notably, our findings revealed that p18 repressed the transcriptional activity of Top1 and GzSNF1, while enhancing that of Hex1. These 20 genes have been established as critical factors influencing the growth, development, sexual and asexual sporulation, and pathogenicity of *F. graminearum* (Table S4). Consequently, we posit that p18 modulates the expression of key host genes at the transcript level, thereby inhibiting viral accumulation and ultimately facilitating viral asymptomatic infection and vertical transmission.

Total RNA was extracted from strains cultured for 4 days, and quantitative reverse transcriptase PCR (RT-qPCR) was performed using EF-1α transcript levels serving as an internal control (n = 3). The experiment was repeated three times with similar results. Representative images are shown. Error bars represent standard deviation. Different letters (a, b, and c) indicate significant differences ($p < 0.05$ determined by Tukey's post hoc test).

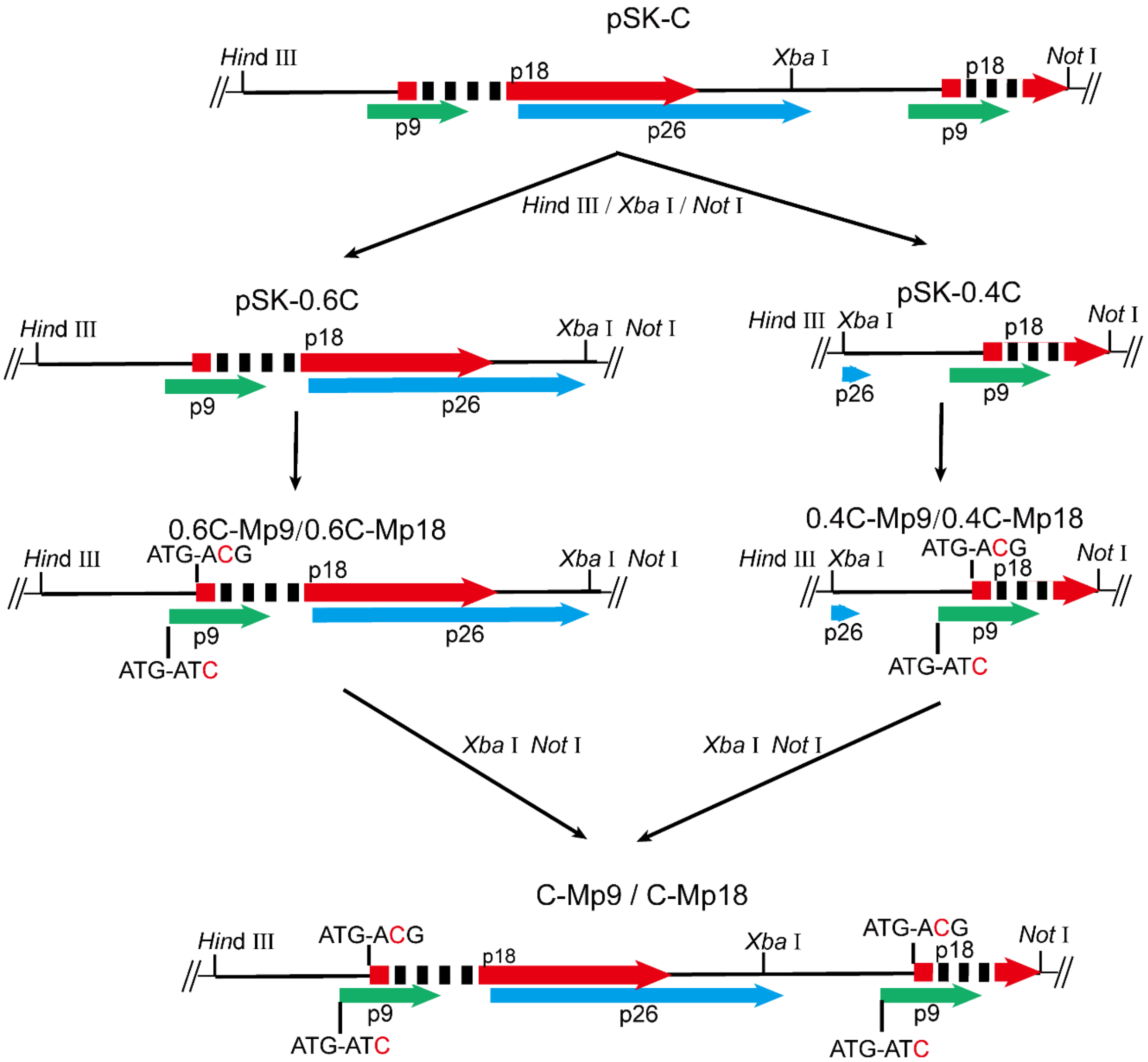

Fig. S9. Construction process of p9 and p18 mutant. The arrows indicate the orientation of the open reading frames (ORFs), with the green arrow signifying the p9 protein, the red arrow the p18 protein, and the blue arrow the p26 protein. The clone pSK-1C was subjected to digestion with *Hin*d III, *Xba* I, and *Not* I enzymes, resulting in the isolation of approximately 1.28 kb and 0.65 kb fragments. These fragments were then inserted into the vector pSK, which had been digested with *Hin*d III/*Xba* I and *Xba* I/*Not* I respectively, yielding the clones pSK-0.6C and pSK-0.4C. Site-directed mutagenesis of 0.6C-Mp9 and 0.4C-Mp9 was carried out using complementary primer pairs and the KOD-Plus-Mutagenesis Kit (Toyobo, Osaka, Japan), with the plasmids pSK-0.6C and pSK-0.4C serving as templates. Following this, the plasmid 0.4C-Mp9 was digested with *Hin*d III and *Xba* I enzymes, and the resulting ~0.65 kb fragment was inserted into the corresponding *Hin*d III and *Xba* I sites of the plasmid 0.6C-Mp9, generating the clone C-Mp9. Finally, C-Mp9 was ligated back into the pSK-AB vector, yielding the construct named Mp9. Mp18 was constructed using a similar approach.

Table S1. All primers used in this study.

Primer name	Primer sequence	Reference
P18-Pro-F	AGGGAACAAAAGCTGGGTACCATCTTTTTTTTTTACAAATCATAACAATTC	Used for p18(500)-GFP construction
P18-Pro-R	GCCCTTGCTCACCATTCTAGATTTGAAAAAGTGTTGGAGTGGTTG	
P26-Pro-F	AGGGAACAAAAGCTGGGTACCAAATTGCGGGTAACATTCCAAT	Used for p26(500)-GFP construction
P26-Pro-R	CCCTTGCTCACCATTCTAGACTTTATATTGTAAAAAATATTTGTAACTGTAAATA	
P9-RT-F	ATGACACCACCAAACATGTCC	Used for p9 full-length amplification
P9-RT-R	TTAAGTAAGGGTCATTGCACCATC	
P18-RT-F	ATGTCTCAATCATCAATTCCAAG	Used for p18 full-length amplification
P18-RT-R	TTAGTGCTGGTATGAACTCGCAGG	
P26-RT-F	ATGTCGACCCAAAACAATCAG	Used for p26 full-length amplification
P26-RT-R	TTAAATTGGAGCATTGTCTAAATC	
Fg-18S-F	AGGCAATAACAGGTCTGTGATGCC	Used for RT-PCR
Fg-18S-R	TGAGCCATTCAATCGGTAGTAGCG	
p9-MUT-F	CAAGGACATGTTTGGTGGTGTGATTGGTTAATATAGGTACTGTCGGTAG	Used for Mp9 construction
p9-MUT-R	CTACCGACAGTACCTATATTAACCAATCACACCACCAAACATGTCCTTG	
p18-MUT-F	CCACTCCAACACTTTTTCAAAACGTCTCAATCATCAATTCCAAGAAC	Used for Mp18 construction

p18-MUT-R	GTTCTTGGAATTGATGATTGAGACGTTTTGAAAAAGTGTTGGAGTGG	
P26-MUT-R	GTTACAAATATTTTTTACAATATAAAGACGTCGACCCAAAACAATCAG	Used for Mp26 construction
P26-MUT-F	CTGATTGTTTTGGGTCGACGTCTTTATATTGTAAAAAATATTTGTAAC	
qPCR-A-F	TGGGAAGTAGGCGTGATT	Used for qPCR amplification
qPCR-A-R	AGCACATACGAATCTCCAC	
qPCR-B-F	GGCAATCCGCAAACACAT	Used for qPCR amplification
qPCR-B-R	GACATATCACGAACCGCCAA	
qPCR-C-F	GCCACAAGCACCTCTTTAGA	Used for qPCR amplification
qPCR-C-R	TCGCCGGTTACAATACAAG	
DNA-EF1α-F	GCTTACTGCCTCCACCAACT	Used for qPCR amplification
DNA-EF1α-R	CGTTCCAATACCGCCAAT	
p18Flag-F	CCTGCGAGTTCATACCAGCACGACTACAAAGACCATGACGGTGATTATAAAGATCATGACATCGACTACAAGGATGACGATGACAAGATGCATTGATCCTGATTGGGTGGACATACCAG	Used for p18-Flag construction
p18Flag-R	CTGGTATGTCCACCCAATCAGGATCAATGCATCTTGTCATCGTCATCCTTGTAGTCGATGTCATGATCTTTATAATCACCGTCATGGTCTTTGTAGTCGTGCTGGTATGAACTCGCAGG	
OE-p18-GFP-F	GTCGACGGTATCGATAAGCTTATGTCTCAATCATCAATTCCAAGAAC	Used for OE-p18-GFP construction
OE-p18-GFP-R	CAGCTCCTCGCCCTTGCTCACGTGCTGGTATGAACTCGCAGG	
OE-p18-Pro-F1	AGGGAACAAAAGCTGGGTACCATCTTTTTTTTTTACAAATCATAAC	

OE-p18-Pro-R1	CTTGGAATTGATGATTGAGACATTTTGAAAAAGTGTTGGAGTGGTTG	
OE-p18-Flag-F1	CAACCACTCCAACACTTTTTCAAAATGTCTCAATCATCAATTCCAAG	Used for OE-p18-Flag construction
OE-p18-Flag-R1	TCACCGTCATGGTCTTTGTAGTCGTGCTGGTATGAACTCGCAGGAG	
qPCR-FgSCH9-F	GTAGTGAGCTCATCTCGGGC	Used for qPCR amplification
qPCR-FgSCH9-R	AATTCTGCTTCCGCATCCCA	
qPCR-FgCPK1-F	TTGGTTTGCCGAAGTCACCT	Used for qPCR amplification
qPCR-FgCPK1-R	ATCATTGACGTCCCACCCAC	
qPCR-FgHtf1-F	ACAACGCTCGCATCAACAAC	Used for qPCR amplification
qPCR-FgHtf1-R	CAGACAGAGGTTCAGGCGTT	
qPCR-Mgv1-F	TGAGGACGTTGGTCAGATGC	Used for qPCR amplification
qPCR-Mgv1-R	ATCCAGGCCTCCCTGAAGAT	
qPCR-Gpmk1-F	CCCCGGAAAGGATTACCACC	Used for qPCR amplification
qPCR-Gpmk1-R	GGCTCAAGGTATGGGTGCTT	
qPCR-Ras2-F	ACGACCCCACGATTGAAGAC	Used for qPCR amplification
qPCR-Ras2-R	GCCAGGATAAGATGGGGACG	
qPCR-Epl3-F	ACGCGACATCAACCTCAACT	Used for qPCR amplification
qPCR-Epl3-R	GCCTGCTCGAATGGATCGTA	
qPCR-Hdf1-F	ACAACGCTCGCATCAACAAC	Used for qPCR amplification
qPCR-Hdf1-R	CAGACAGAGGTTCAGGCGTT	

qPCR-GzmetE-F	GCAGCAGCTAGCGATTGTTC	Used for qPCR amplification
qPCR-GzmetE-R	GACATCGTCGCGAATGGTTG	
qPCR-FgCch1-F	CGAGTACCGATTACGCCGAA	Used for qPCR amplification
qPCR-FgCch1-R	ACCTCAGTCTCTCCGCTGAT	
qPCR-Mid1-F	GCAGAACAGTCCCTCCTGAC	Used for qPCR amplification
qPCR-Mid1-R	GAGTTGTGGCGCTGTTTCAG	
qPCR-Fig1-F	AAGGTCCAGGTCGGATGGTA	Used for qPCR amplification
qPCR-Fig1-R	GAACCACCATCGGGGTTGAT	
qPCR-Gea1-F	AGAAAACCACAGCGACGGAT	Used for qPCR amplification
qPCR-Gea1-R	GGTGGAAGCGGAAGTGCTAT	
qPCR-Tub1-F	AGCTCACCCAGCAGATGTTC	Used for qPCR amplification
qPCR-Tub1-R	TCGCCAACACGCTTGAAAAG	
qPCR-FgPal1-F	TCAAGCAAAACAGCAAGCCC	Used for qPCR amplification
qPCR-FgPal1-R	TTTACGTCGTCTGGCAGCTT	
qPCR-FgFlbD-F	AACCACGAACCCATCTCACC	Used for qPCR amplification
qPCR-FlbD-R	GCGTGATAGGACCCGAGATG	
qPCR-StuA-F	CAAGGGCGTCTGGATTCCTT	Used for qPCR amplification
qPCR-StuA -R	AACCATTCATGGGAGGCTGG	
qPCR-Top1-F	AAGGTGTTCCACTGGCCAAA	Used for qPCR amplification

qPCR-Top1-R	CTTCGCGGTTCCGTTTGTTT	
qPCR-Hex1-F	TCGAGATTGACATCCGCGAG	Used for qPCR amplification
qPCR-Hex1-R	ATGGACTGAACGACAACGCT	
qPCR-GzSNF1-F	AGCAAGGTTGGTATCCTGCC	Used for qPCR amplification
qPCR-GzSNF1-R	GAGTTGCGTGATCGAATGCC	
RNA-EF1α-F	GAAGTTCGAGAAGGAAGC	Used for qPCR amplification
RNA-EF1α-R	ATGACGGTGACATAGTAG	

Table S2. All *F. graminearum* strains used in this study.

Strains	Brief description	Reference
PH-1	Wild-type	(53)
PH-1^{neo}	PH-1 integrated the neomycin-resistance gene (*neo*)	(34)
PH-1(WT)::pSK-ABC	Transfectants of the infectious clone pSK-ABC, strain PH-1(WT) as a recipient	(34)
PH-1(WT)::pSK-AB	Transfectants of the infectious clone pSK-AB, strain PH-1(WT) as a recipient	This study
p26(500bp)-GFP	Transformants for detecting the activity of p26 promoter, strain PH-1(WT) as a recipient	This study
p18(500bp)-GFP	Transformants for detecting the activity of p18 promoter, strain PH-1(WT) as a recipient	This study
RP27-GFP	PH-1(WT) integrated RP27 promoter to express enhanced green fluorescent protein (eGFP)	This study
PH-1(WT)::Mp9	Transfectants of the viral deletion mutant Mp9, strain PH-1(WT) as a recipient	This study
PH-1(WT)::Mp18	Transfectants of the viral deletion mutant Mp18, strain PH-1(WT) as a recipient	This study
PH-1(WT)::Mp26	Transfectants of the viral deletion mutant Mp26, strain PH-1(WT) as a recipient	This study
PH-1(WT)::Mp9:p18	Transfectants of the viral deletion mutant Mp9:p18, strain PH-1(WT) as a recipient	This study
PH-1(WT)::Mp9:p26	Transfectants of the viral deletion mutant Mp9:p26, strain PH-1(WT) as a recipient	This study
PH-1(WT)::Mp18:p26	Transfectants of the viral deletion mutant Mp18:p26, strain PH-1(WT) as a recipient	This study
PH-1(WT)::Mp9:p18:p26	Transfectants of the viral deletion mutant Mp9:p18:p26, strain PH-1(WT) as a recipient	This study
PH-1(WT)::p18-Flag	Transfectants of the viral deletion mutant pSK-ABC::p18-Flag, strain PH-1(WT) as a recipient	This study
PH-1(WT)::Mp18-Flag	Transfectants of the viral deletion mutant pSK-ABC::Mp18-Flag, strain PH-1(WT) as a recipient	This study
OE-p18-GFP	Transformants for detecting the localization of p18 protein, strain PH-1(WT) as a recipient	This study

OE-p18-Flag	Transformants for overexpressing p18 protein, strain PH-1(WT) as a recipient	This study
OE-p18-Flag::pSK-AB	Transfectants of the infectious clone pSK-AB, strain OE-p18-Flag as a recipient	This study
OE-p18-Flag::Mp18	Transfectants of the viral deletion mutant Mp18, strain OE-p18-Flag as a recipient	This study

Table S3. The unique p18 polypeptide in FgGMTV1-infected strains was detected by mass spectrometry.

Sequence	Length	Mass	Unique	PEP	Score
FEETHPTLLQR	11	1369.699	yes	0.0249	9.5142
MSQSSIPRTYK	11	1296.649	no	1.0	none
SQSSIPRTYK	10	1165.609	no	1.0	none
YFLQYKDVDPK	11	1414.713	yes	0.0177	9.5142

Note: Sequence: The amino acid sequence of the identified peptide. Length:The length of the sequence, as stored in the "Sequence" column. Mass: the monoisotopic mass of the peptide. Unique: When marked with 'yes', this particular peptide is unique to a single protein group in the protein groups file. PEP: Posterior Error Probability of the identification. This value essentially operates as a p-value, where a lower value indicates greater significant. Score: The Andromeda score for the best associated MS/MS spectrum.

Table S4. The genes used to detect transcriptional levels in this work.

Gene name	Brief description	Reference
FgSCH9 (FGSG_00472)	*FgSCH9* is orthologous to *SCH9* and is functionally related to the cAMP-PKA and TOR pathways in the budding yeast. *FgSCH9* is important for stress responses, DON production, conidiogenesis, and pathogenesis in *F. graminearum*.	(1)
CPK1 (FGSG_07251)	CPK1 encodes the catalytic subunits of cyclic AMP (cAMP)-dependent protein kinase A (PKA), with the cAMP-PKA pathway playing critical roles in hyphal growth, conidiation, ascosporogenesis, and plant infection in *F. graminearum*.	(2)
FgHtf1 (FGSG_07097)	*FgHtf1*, a transcription factor, controls global gene expression and promotes a shift to aerial growth and conidiation in *F. graminearum* by activating of genes related to conidiation.	(3,4)
Mgv1 (FGSG 10313)	Mgv1, one of the MAP kinase genes, is involved in regulating hyphal growth, sexual reproduction, plant infection, and stress responses in *F. graminearum*.	(5)
Gpmk1 (FGSG_06385)	*Gpmk1* is responsible for signal transduction processes taking place during the most important developmental stages in the life cycle of this fungal pathogen.	(6)
Ras2 (FGSG_10114)	RAS2 encode Ras GTPases, which are dispensable for survival but, when disrupted, elicit a variety of morphological defects, including slower growth on solid media, delayed spore germination, and significant reductions in virulence on wheat heads and maize silks. *Ras2* regulates growth and virulence by regulating the *Gpmk1* MAP kinase pathway in *F. graminearum*.	(7)
Epl3 (FGSG_02040)	EPL3, an ortholog of yeast elongator complex protein 3, functions in sexual and asexual development, virulence, and the oxidative stress response in *F. graminearum* by regulating the expression of genes involved in these various developmental processes.	(8)
Hdf1 (FGSG_01353)	*Hdf1*, an orthologue of yeast HOS2, represents the primary class II histone deacetylase (HDAC) gene in *F. graminearum.* It functions as a component in a well-conserved HDAC complex in the regulation of conidiation, DON production, and pathogenesis.	(9)
GzmetE (FGSG_05658)	GzmetE encodes a homoserine O-acetyltransferase, which is important for sexual development and plant infection.	(10)

Cch1 (FGSG_01364)	*Cch1* is a putative voltage-gated calcium ion channel and has subtle effects on growth and development. In the *Cch1* deleted mutant, mycelial growth was significantly slower, and sexual development was slightly delayed, mutant mycelia showed a distinctive fluffy morphology, and no cirrhi were produced.	(11)
Mid1 (FGSG_07418)	*Mid1* acts in a stretch-activated ion channel capable of being permeated by calcium. *Mid1* deleted mutants exhibited significantly reduced forcible ascospore discharge, slowed vegetative growth, reduced conidiation, and a high frequency of abnormal ascospores.	(12)
Fig1 (FGSG_06302)	*Fig1* is a transmembrane protein of the low-affinity calcium uptake system (LACS) in fungi. The *Fig1* deleted mutants failed to produce mature perithecia, with sexual development halted before the formation of perithecium initials. The loss of *Fig1* function also resulted in a reduced vegetative growth rate.	(13)
Gea1 (FGSG_08505)	*Gea1* is required for the development of the ascus wall. *Gea1* deletion mutants produced normal-shaped perithecia and ascospores, yet ascospores prematurely germinated inside the perithecium. Moreover, *Gea1* deletions resulted in abnormal ascus walls that collapsed before ascospore discharge.	(14)
Tub1 (FGSG 09530)	*TUB1* plays an essential role in ascosporogenesis and sexual-specific functions of *TUB1* require stage-specific RNA processing and Tub1 expression.	(15, 16)
FgPal1 (FGSG_08726)	*FgPal1* plays a role in maintaining polarized tip growth and coordination between nuclear division and cytokinesis, and it is also important for infectious growth and development of ascospores by the free cell formation process.	(17)
FlbD (FGSG_01915)	*FlbD*, an orthologue of the fluffy gene of *Aspergillus nidulans*, is the only conserved regulator for conidiogenesis in *F. graminearum*. Deletion of *FgFlbD* prevented hyphal differentiation and the formation of perithecia.	(18)
StuA (FGSG_10129)	*StuA* is a transcription factor with homology to key developmental regulators in fungi. The deletion mutant exhibited severely diminished pathogenicity on wheat heads and secondary metabolite production. Spore production was significantly impaired in ΔFgStuA, which did not develop perithecia and sexual ascospores, and lacked conidiophores and phialides, leading to delayed production of aberrant macroconidia.	(19)

Top1 (FGSG_06874)	The lack of *Top1* (topoisomerase 1) enzyme activity causes severe reductions in the ability of fungal mycelium to colonize wheat ear tissues post penetration.	(20)
Hex1 (FGSG_08737)	The HEX1 protein is the major constituent of the Woronin body (WB), which is a peroxisome-derived electron-dense core organelle that seals the septal pore in response to hyphal wounding.	(21, 22)
GzSNF1 (FGSG_09897)	SNF1 (sucrose nonfermenting 1) protein kinase is critical for normal sexual and asexual development, in addition to virulence and the utilization of alternative carbon sources.	(23)

REFERENCES

1. Wilson RA, Chen D, Wang Y, Zhou X, Wang Y, Xu J-R. 2014. The Sch9 kinase regulates conidium size, stress responses, and pathogenesis in Fusarium graminearum. PLoS ONE 9.
2. Hu S, Zhou X, Gu X, Cao S, Wang C, Xu J-R. 2014. The cAMP-PKA pathway regulates growth, sexual and asexual differentiation, and pathogenesis in Fusarium graminearum. Mol Plant Microbe In 27:557-566.
3. Chen S, Li P, Abubakar YS, Lü P, Li Y, Mao X, Zhang C, Zheng W, Wang Z, Lu G-d, Zheng H. 2024. A feedback regulation of FgHtf1-FgCon7 loop in conidiogenesis and development of Fusarium graminearum. Int J Biol Macromol 261.
4. Fan GL, Zheng HW, Zhang K, Ganeshan VD, Opiyo SO, Liu D, Li MY, Li GP, Mitchell TK, Yun YZ, Wang ZH, Lu GD. 2020. FgHtf1 regulates global gene expression towards aerial mycelium and conidiophore formation in the cereal fungal pathogen. Appl Environ Microb 86.
5. Ren J, Li C, Gao C, Xu J-R, Jiang C, Wang G. 2019. Deletion of FgHOG1 is suppressive to the mgv1 mutant by stimulating Gpmk1 activation and avoiding intracellular turgor elevation in Fusarium graminearum. Front Microbiol 10.
6. Jenczmionka NJ, Maier FJ, Lösch AP, Schäfer W. 2003. Mating, conidiation and pathogenicity of Fusarium graminearum, the main causal agent of the head-blight disease of wheat, are regulated by the MAP kinase gpmk1. Curr Genet 43:87-95.
7. Bluhm BH, Zhao X, Flaherty JE, Xu JR, Dunkle LD. 2007. RAS2 regulates growth and pathogenesis in Fusarium graminearum. Mol Plant Microbe Interact 20:627-36.
9. Li Y, Wang C, Liu W, Wang G, Kang Z, Kistler HC, Xu JR. 2011. The HDF1 histone deacetylase gene is important for conidiation, sexual reproduction, and pathogenesis in Fusarium graminearum. Mol Plant Microbe Interact 24:487-96.
8. Lee Y, Min K, Son H, Park AR, Kim J-C, Choi GJ, Lee Y-W. 2014. ELP3 is involved in sexual and asexual development, virulence, and the oxidative stress response in Fusarium graminearum. Mol Plant Microbe In 27:1344-1355.
10. Wang J, Zeng W, Xie J, Fu Y, Jiang D, Lin Y, Chen W, Cheng J. 2021. A novel antisense long non‐coding RNA participates in asexual and sexual reproduction by regulating the expression of GzmetE in Fusarium graminearum. Environ Microbiol 23:4939-4955.
11. Hallen HE, Trail F. 2008. The L-Type calcium ion channel Cch1 affects ascospore discharge and mycelial growth in the filamentous fungus Gibberella zeae (anamorph Fusarium graminearum). Eukaryotic Cell 7:415-424.
12. Cavinder B, Hamam A, Lew RR, Trail F. 2011. Mid1, a mechanosensitive calcium ion channel, affects growth, development, and ascospore discharge in the filamentous fungus Gibberella zeae. Eukaryotic Cell 10:832-841.
13. Cavinder B, Trail F. 2012. Role of Fig1, a component of the low-affinity calcium uptake system, in growth and sexual development of filamentous fungi. Eukaryotic Cell 11:978-988.

14. Son H, Lee J, Lee Y-W. 2013. A novel gene, GEA1, is required for ascus cell-wall development in the ascomycete fungus Fusarium graminearum. Microbiology 159:1077-1085.
15. Chen D, Wu C, Hao C, Huang P, Liu H, Bian Z, Xu JR. 2018. Sexual specific functions of Tub1 beta‐tubulins require stage‐specific RNA processing and expression in Fusarium graminearum. Environ Microbiol 20:4009-4021.
16. Wang H, Chen D, Li C, Tian N, Zhang J, Xu J-R, Wang C. 2019. Stage-specific functional relationships between Tub1 and Tub2 beta-tubulins in the wheat scab fungus Fusarium graminearum. Fungal Genet Biol 132.
17. Yin J, Hao C, Niu G, Wang W, Wang G, Xiang P, Xu JR, Zhang X. 2020. FgPal1 regulates morphogenesis and pathogenesis in Fusarium graminearum. Environ Microbiol 22:5373-5386.
18. Son H, Kim M-G, Chae S-K, Lee Y-W. 2014. FgFlbD regulates hyphal differentiation required for sexual and asexual reproduction in the ascomycete fungus Fusarium graminearum. J Microbiol 52:930-939.
19. Lysøe E, Pasquali M, Breakspear A, Kistler HC. 2011. The transcription factor FgStuAp influences spore development, pathogenicity, and secondary metabolism in Fusarium graminearum. Mol Plant Microbe In 24:54-67.
20. Baldwin TK, Urban M, Brown N, Hammond-Kosack KE. 2010. A role for topoisomerase I in Fusarium graminearum and F. culmorum pathogenesis and sporulation. Mol Plant Microbe Interact 23:566-77.
21. Son M, Lee KM, Yu J, Kang M, Park JM, Kwon SJ, Kim KH. 2013. The HEX1 gene of Fusarium graminearum is required for fungal asexual reproduction and pathogenesis and for efficient viral RNA accumulation of Fusarium graminearum virus 1. J Virol 87:10356-67.
22. Son M, Choi H, Kim KH. 2016. Specific binding of Fusarium graminearum Hex1 protein to untranslated regions of the genomic RNA of Fusarium graminearum virus 1 correlates with increased accumulation of both strands of viral RNA. Virology 489:202-11.
23. Lee S-H, Lee J, Lee S, Park E-H, Kim K-W, Kim M-D, Yun S-H, Lee Y-W. 2009. GzSNF1 is required for normal sexual and asexual development in the ascomycete Gibberella zeae. Eukaryotic Cell 8:116-127.
